# Conservation and Dispersion of Genes Conferring Resistance to Tomato Begomoviruses between Tomato and Pepper Genomes

**DOI:** 10.3389/fpls.2017.01803

**Published:** 2017-11-07

**Authors:** Manisha Mangal, Arpita Srivastava, Rita Sharma, Pritam Kalia

**Affiliations:** ^1^Division of Vegetable Science, ICAR-Indian Agricultural Research Institute, New Delhi, India; ^2^Crop Genetics and Informatics Group, School of Computational and Integrative Sciences, Jawaharlal Nehru University, New Delhi, India

**Keywords:** begomoviruses, ChLCV, pepper, tomato, TYLCV, genome conservation

## Abstract

In the present climate change scenario, controlling plant disease through exploitation of host plant resistance could contribute toward the sustainable crop production and global food security. In this respect, the identification of new sources of resistance and utilization of genetic diversity within the species may help in the generation of cultivars with improved disease resistance. Begomoviruses namely, *Tomato yellow leaf curl virus* (TYLCV) and *Chilli leaf curl virus* (ChLCV) are known to cause major yield losses in several economically important crop plants of the family Solanaceae. Though co-occurrence, association and synergistic interactions among these viruses in the host plants is reported, whether orthologous genetic loci in related host plants could be responsible for conferring resistance to these viruses has not been investigated yet. Several loci including *Ty1, Ty2, Ty3, Ty4*, and *ty5* have been reported to confer resistance to leaf curl viruses in tomato. Here, we examined the pepper orthologous markers, corresponding to these QTL regions, for polymorphism between ChLCV susceptible and resistant genotypes of pepper. Further, to examine if the polymorphic markers are segregating with the disease resistance, Bulk Segregant Analysis (BSA) was performed on F_2_ population derived from crosses between resistant and susceptible lines. However, none of the markers showed polymorphism in BSA suggesting that the tested markers are not linked to genes/QTLs responsible for conferring resistance to ChLCV in the selected genotypes. *In silico* analysis was performed to study the synteny and collinearity of genes located within these QTL regions in tomato and pepper genomes, which revealed that more than 60% genes located in *Ty2* and *Ty4*, 13.71% genes in *Ty1*, 23.07% in *Ty3*, and 44.77% genes located within *ty5* QTL region in tomato are conserved in pepper genome. However, despite such a high conservation in gene content, the linkage relationship in these regions seems to be greatly affected by gross rearrangements in both the species.

## Introduction

Hot pepper (*Capsicum annuum L*.) is extensively cultivated throughout the world, as an essential condiment and a cash crop (Bosland and Votava, 2000). In India, pepper is used as a vegetable, spice as well as for industrial purposes involving extraction of oleoresin and capsaicin (Kumar and Rai, 2005). Although pepper is infested by a large number of pathogens, viruses in-particular, cause heavy losses both in terms of quality and productivity of pepper. Whitefly (*Bemisia tabaci* Genn.) transmitted begomovirus ChLCV has been reported to cause havoc in the hot pepper growing areas of North India covering states of Delhi, Haryana, Rajasthan, Punjab, West Bengal, and Uttar Pradesh with up to 100% crop loss (Senanayake et al., 2007). Similar severity has also been reported in the major hot pepper growing districts of Andhra Pradesh in South India. The typical symptoms under field conditions include upward curling, puckering, smaller leaves, stunted stems, and lack of fruiting. Furthermore, several other begomoviruses including *Chilli leaf curl India virus, Tomato leaf curl Joydebpur virus* and *Tomato leaf curl New Delhi virus* (ToLCNDV) have been found to co-infect the pepper plants along with ChLCV under field conditions (Khan et al., 2006; Fortes et al., 2016; Srivastava et al., 2017). In fact, synergistic interactions between different begomoviruses infecting pepper have been reported to cause breakdown of natural resistance in the host plant (Singh et al., 2016; Al-Shihi et al., 2017).

At Division of Vegetable Science, ICAR-IARI, New Delhi, we had earlier tested 62 germplasm lines of hot pepper for resistance to the ChLCV under natural disease epiphytotic conditions in Trans-Gangetic plains of Northern India. In addition to field screening, the germplasm of hot pepper was subjected to virus indexing against ChLCV as well as ToLCNDV. The variables measured included disease incidence and severity. Scales for classifying the lines tested for leaf curl disease reactions were adopted from Kumar et al. (2006). Two genotypes of hot pepper namely, DLS-Sel-10 and WBC–Sel-5, were found resistant to ChLCV infection (Srivastava et al., 2017). Also, we observed, during these trials, that ToLCNDV infection was commonly prevalent along with ChLCV infection in the field conditions. However, resistant genotypes, DLS-Sel-10 and WBC-Sel-5, showed no incidence of any of the two viruses, indicating that these lines are likely resistant to ToLCNDV as well (Srivastava et al., 2015). The genetic basis of this resistance is yet to be examined.

*Ty* QTLs of tomato have been reported to confer resistance against both monopartite and bipartite tomato leaf curl viruses, prevalent in India (Ji et al., 2007b; Anbinder et al., 2009; Prasanna et al., 2015; Fortes et al., 2016). The *Ty1* locus, mapped on chromosome 6 from *Solanum chilense* LA1969 (Zamir et al., 1994), was later reported to be linked to a major QTL *Ty3* that has 60% contribution in symptom severity (Ji et al., 2007a). *Ty2*, first detected in *S. habrochaites* B6013, was mapped to chromosome 11 (Hanson et al., 2006; Ji et al., 2009a; Yang et al., 2014). *Ty4*, that accounts for only 16% of the variation, was located on the long arm of chromosome 3 (Ji et al., 2009b). A line derived from *S. peruvianum* was also reported to be the source of recessive resistance and the QTL responsible for resistance was named as *ty5* (Friedmann et al., 1998; Anbinder et al., 2009). The *ty*5, mapped to chromosome 4, has been predicted to have originated from cultivar Tyking (Hutton et al., 2012). Hutton and Scott (2013) mapped another recessive resistance gene *ty6* to chromosome 10, in *Ty*3-carrying *S. chilense* “LA2779”. Although initially these QTLs were identified as source of resistance to *Tomato yellow leaf curl virus* (TYLCV), recent studies reported that *Ty3* is highly effective against monopartite *Tomato leaf curl Bangalore virus* (ToLCBV) as well as two bipartite viruses, ToLCNDV and *Tomato leaf curl Palampur virus* from India (Prasanna et al., 2015). Similarly, *Ty1* gene has been shown to be effective against ToLCBV and ToLCNDV (Prasanna et al., 2015). Since the resistant lines selected in our study had shown resistant response to both ChLCV as well as ToLCNDV, we initiated this study to check if pepper genes orthologous to *Ty* loci of tomato, could be responsible for conferring resistance to ChLCV. We analyzed polymorphism in orthologous markers located within the QTL regions of *Ty1, Ty2, Ty3, Ty4*, and *ty5* between a ChLCV-sensitive and -resistant genotype of hot pepper. Polymorphic markers between resistant and susceptible parents were then used for Bulk Segregant Analysis (BSA) on F_2_ population in order to find out if any of the markers is segregating with the resistance trait. Furthermore, *in silico* analysis was carried out to study the conservation and dispersion of genes within *Ty* QTL regions between tomato and pepper genomes.

## Materials and methods

### Extraction of orthologous markers in pepper and tomato genomes

We used conserved ortholog set II (COSII) markers provided by Wu et al. (2009) for extracting the orthologous markers in and around *Ty* QTL regions between tomato and pepper genomes. Since the COSII markers mapped in this study provide a detail inference of syntenic regions between tomato and pepper genomes, we identified all the markers flanking *Ty QTLs* of tomato on genetic map of pepper and extracted all the markers located between and adjacent to these markers in pepper genome for further analysis. In case, the orthologs of flanking markers of *Ty* QTL of tomato were located on two different chromosomes of pepper, all the markers flanking these orthologous markers from both the chromosomes of pepper were extracted for the downstream analysis. The complete list of markers flanking *Ty* QTLs of tomato and list of orthologous markers extracted in pepper for further analysis are provided in Tables 1 and 2, respectively.

**Table 1:** Markers flanking *Ty* QTLs of tomato and their physical locations.

**Name of QTL**	**Linkage group**	**Flanking marker(s) on one side of QTL**		**Flanking marker(s) on the other side of QTL**		**Reference for markers**
		**Name of marker**	**Location on chromosome (bp)**	**Name of marker**	**Location on chromosome (bp)**	
Ty1	6	C2_At5g61510	23699998	C2At3g10920/T1456	29685557	Ji et al., 2007a
Ty2	11	C2_AT1g07960	54406711	T0302	54795529	Ji et al., 2009a
Ty3	6	C2_At5g05690	35309536	C2_At5g41480	35582868	Ji et al., 2007a
		To507	35310220	T0693	35582868	
Ty4	3	C2_AT4g17300	61277283	C2_AT5g60160	61828034	Ji et al., 2009b
		T1320	61281818			
Ty5	4	SSR43	2127724	TG182	4834081	Anbinder et al., 2009; Kadirvel et al., 2013

**Table 2:** List of orthologous markers used for validation in pepper.^*^

	**Marker name**	**Linkage group**	**Map unit (cM)**	**Marker type**	**(d)CAPS enzyme**	**Forward primer**	**Reverse primer**
1.	C2At1g05385	3	41.5	dCAPS	AccI	ACGAACAGCTGATGCAGCAAAGG	GACCAGATGAAACAAACTCAGGTAG
2.	C2At2g47580	3	46.5	CAPS	Hpy188I	TAGCGGCGGCGAAGTTCCAC	ATCAAACACTACCCACGCCTGTCC
3.	C2At4g03200	3	50.9	CAPS	RsaI	TGGGGCTGAGCCTTCAGGGAA	GCCAGCATATCTGCTGCACAGCAC
4.	C2At5g42740	3	53	PCR	NA	AGCACCATTTGAGAAAAATATACCTG	ATCCAAGGAATGAAACATTCCACAC
5.	TG517	3	55.1	dCAPS	BclI	CTTCTTCTGCTCAGCAGCAACATCATGA	TTTATCGGCTCTCGTTTTGC
6.	C2At4g18593	3	59.3	CAPS	AluI	AGGTGATTGTTATAATCGTGGAGAAAG	TTCACAATGCGCACATAAAAGCTTG
7.	C2At1g64770	3	61.4	CAPS	BstBI	TCCGGAGCTGTACTTATTGCACACATC	AGCCCAAACGTATTATCCTAAAGAAGAG
8.	C2At3g13180	3	61.4	dCAPS	RsaI	TATTGTTGATCCACAGCCTGGTGAG	AGGGAATATGGTGCTTGTATTGAAGG
9.	C2At5g62390	3	63.3	CAPS	TaqI	TGCTACTAACTGTTGATGCCATTGAG	TTGGGGGTCGATAACATCAAGC
10.	C2At3g63530	3	67.1	dCAPS	CfoI	CTTGTGTTAGCATTTAGAAAAACTGCG	AACATGTGTGCACAAGGTAAAGTGGTC
11.	C2At5g23880	3	71.1	CAPS	RsaI	AGCTAACCTAATCCTTGATACAACACC	ACCATCAGAACGACCTTCGAAGTCC
12.	C2At1g51160	3	75.1	CAPS	DraI	TCTTCGGAGGATCAGAGATCAGTCC	AACGAACATCCTTGTCCAGGTAATTG
13.	C2At5g17170	3	91.9	dCAPS	RsaI	TTCAAGGGCTATCATTACAAGAGGC	CTTGCGAGAAATTCTCTAATAAGTGGT
14.	C2At3g03100	3	94.1	CAPS	AflII	TGGTGCAACACTTGTTGGTGTGG	TGGAGCCAGCCATGCCATTC
15.	C2At5g41040	3	95.8	CAPS	HinfI	AGAAGGGGCTGTCTTTGTTGAGGC	TCGCGCTTTCCAGACGAAAGCTG
16.	C2At5g52820	3	109.5	CAPS	AseI	TGGGATCTAAATACCCAGACACC	ACAGAAAGAACCCAATTTCTGTGC
17.	C2At5g49970	3	114.4	CAPS	EcoRV	AATTGGCAGGCTTGAGTGTTGC	TCCCACCATTGTTACCAGGACCAC
18.	C2At5g23060	3	119.2	CAPS	AseI	ACTTAGAGCTTCTTCAGCCACCGC	ATGCCAGCACTCTGCATTGCCTC
19.	C2At1g18660	3	123.9	CAPS	TaqI	ACCCTGTGCTTAAAGTTATTATATGAACC	ACTGTTCGGCACAATGGACATCTG
20.	C2At1g80170	3	125.3	CAPS	PvuII	TTTAACTTTCCACAAATGCAACAACC	TCTTTATTCTAACCCCGTTCTCAGTG
21.	C2At3g47640	3	126.7	CAPS	AvaII	AGCTGCTCGATTTGTAAAGGACATGC	TGGCCATACATCATTTGGAGTGGG
22.	C2At1g16180	3	128	CAPS	RsaI	TTCTTGTCTTGCGTCATGCTGTGC	ACCAGCAAATGATTTTCATCATCC
23.	C2At5g42950	4	139.6	dCAPS	SspI	CTCTTCTGGAACACATTATCGTCCCAA	ACATTTTTGGCACTTGCACCAGTGAC
24.	C2At1g75350	4	140.6	CAPS	HincII	AATGTCGCTCCTCCTTCATTCTCC	ATGTATATCCTTCTTCCTGCAGCTCC
25.	C2At1g63610	4	142.8	CAPS	BsiHKAI	TGATGACTGGATATATGTTTAGGAATGC	ACGATTCAATTCCTCCACTTCTGCTTC
26.	C2At1g20575	4	145	CAPS	TaqI	AGACACTACTTTGGCCGGGTGTATC	ATGATGTCTTCTAGTGCAGACTTCTGG
27.	C2At1g42990	4	147.2	CAPS	DpnII	ATGACCCCGTCGATAAGAAGCG	ACCTCACAGCTGCATCTCTATTCCTC
28.	C2At1g76080	4	158.6	CAPS	AluI	TAGTATGGAGGAATTGGATGAAGC	TCTTCTCTGCTGTGGAGCTGCAC
29.	C2At4g25650	5	0	dCAPS	EcoRV	AGCGCAAGCTAAAGGAAATTGG	AAGTCTGTACTGAACAATCTAGCAAGATA
30.	C2At3g52155	5	7.3	dCAPS	StyI	TTCTCATACCCCACTTGTGAGATTCCA	AAGTTGTCCAGATCTACTCAAAGGACG
31.	T0635	5	11.1	CAPS	PstI	TCAACCAACAACAAGGGTCA	CCAGGAGCATCACAGTCAAA
32.	C2At2g39580	5	14.9	CAPS	BsaHI	AGATAACTGGTTTGACGAAGTTCCTGG	TGAAGCTTCTACCCATACATATTCTGG
33.	C2At1g08630	5	18.3	CAPS	BslI	TATTCTCGGTGATTATTCCCATATCC	AGACCATACTTCTTTGCTAGCTCTCC
34.	C2At1g60560	5	19.4	CAPS	BclI	TAGGGAACATAGTGTACAGCATTTGGG	TCAGACTATATGATGATTCACATCTTGG
35.	T0707	5	34.1	CAPS	DpnII	TCGTGGATTATGGGCTTCTT	TCAAAGTTTTATTGATGATGTTCGAC
36.	C2At3g17040	5	36.8	CAPS	AseI	TGGGGTTGGATGGAGTGGAAAG	AGTAGAGGTTACGAATTTCCTCTGC
37.	C2At3g51010	5	39.4	CAPS	HindIII	TCCAAACAATCCCAATGAAGGAAG	ACGCTCTACTCGCTTAATCATTTTC
38.	C2At1g33970	5	44.6	CAPS	BstXI	TGGAAGTGCAATAAGTGATGATTGGG	TCGTTTCCAACAAATTCAGGTTCAG
39.	C2At2g01770	5	49.8	dCAPS	TaqI	ACCATGTATGAAAGGAGTTGTACCTCG	AATTTACAGCAACTTGCATATGGAGA
40.	C2At1g27385	5	53.1	CAPS	HinfI	ACCGTGCATGATGATTCAACTAATGAG	AGTACCAATAGCTGTAAAGCCTCTTTC
41.	C2At2g46580	5	56.4	dCAPS	TaqI	TGCTGGTATTTCACTGAAACTTGGG	TGTTCATCTAGAGAAGGAAGCCCTCG
42.	C2At3g10920	5	70.9	dCAPS	AflII	TGGCTTGGTGTGGACAAAGAGC	GATGAGCACTCATGAAAGTTGGACTTA
43.	U221402	5	72.1	PCR	NA	AAGCCTCCTTGACAAATGCATATAG	AGATATAGCTACAGTGGCAGCTTCATC
44.	C2At3g55800	5	74.5	CAPS	TaqI	ATGCTTGTTCTGAGGAAGTTCCTGAG	AGTTCGTGTCCACAATACTAGAACCATC
45.	C2At3g06440	5	76.8	CAPS	BstNI	TCGTTAGGATTGATGAAGTTCTATCTAGC	GATTCACAACCGGCATTGTAAAATCT
46.	C2At4g12590	5	82.1	dCAPS	HinfI	ACATGGCTATGGATATGATGAAGAAG	ACCAAGCTCTTAATATTGACAAAGAAT
47.	C2At4g01900	6	0	dCAPS	MseI	GAGTTTTAGTGCATCGGACTGCTTTA	TGGGCGCCAAAACCACGAAC
48.	C2At1g72030	6	2.7	CAPS	AvaII	AATGTTGCTGCTGTTCAAGCTGAAGC	ATCACCTGAAACTACTATATAACCTGCAT
49.	C2At2g39690	6	8.8	dCAPS	SspI	TGGTCTTGAATATCCAGAACCTAATG	CTCTGTCTGTTAAATTTGACGAAAAT
50.	C2At3g25120	6	36.8	CAPS	HinfI	CCTTCCTCGGATCGAAAACATT	AGCACTTGGATAGGCGACCATTC
51.	C2At2g30100	6	41.2	PCR	NA	CAAACTATTTCAGATTTACACTTAAATG	ACCGTTCAAGTTGGCTCTTCACAACAG
52.	C2At3g46780	6	46.7	CAPS	ApoI	TGGAGTTTCTGACCTTGGTGCTGC	TCTGCATCTTGAAATGATGATGCAAC
53.	C2At2g29630	6	47.6	CAPS	TaqI	TCTGAGACCTGGTTCAATTTATGATGC	TGGTACGTGTCCAGGCCCTTCATTC
54.	C2At3g56130	6	48.5	dCAPS	CfoI	CTATCTTGTGTATGCCTTGTGAGCAG	AGAGGATTTTCAAGACTTCTCCAGCC
55.	C2At3g56040	6	49.5	CAPS	AccI	TCGCTATTGGATATAATGCGTAATGC	AACTCAGCAACCTCTATTAGCAACTC
56.	C2At1g06110	6	55.9	CAPS	EcoRV	TTGACTTATCTTCTCCAATTGACCC	AGAGAACCCTAGTAGGTAGAGGCAG
57.	C2At1g44760	6	62.2	CAPS	EcoRI	TTCTTCATCTGCTGCTCATCTTGC	AGAGGGTTTTTTCTGACCCAAGAC
58.	C2At1g03150	6	80.6	dCAPS	AluI	TGCCAGTTTCCTGCCGGATTA	AACAATCAACATTACAAATCATATTAGC
59.	C2At1g79810	6	90.4	CAPS	BanII	ATATGAACCAGAACTTGATGCTTTTC	ACGAGCCCATATATATTGACCACCAAC
60.	C2At1g73885	6	94.4	dCAPS	TaqI	TGGTGCACCATCCACAAGGCCA	AGAAAACAAATATAAGTTTTCCCTCGT
61.	C2At5g07960	6	98.4	CAPS	RsaI	AAGATCTTCCTATAGATTACTCCG	TGAATATAATAGCAAGCCACGAGC
62.	C2At1g24360	6	103	CAPS	HaeIII	TCCGGTTGTTATTGTCACTGGAGC	TGGAAACTTCTTCTGCCTCCTTTG
63.	C2At2g43360	6	110.3	PCR	NA	TCGATCTCCTCTTTCATGGCG	TTGAGGACAATACGAACAATCTTC
64.	C2At2g27450	11	91.9	dCAPS	RsaI	TCAAGATGATGGACTTGATTCTCG	AGAGAAAATGTTACATTTGCTAGTA
65.	C2At3g44600	11	99.2	CAPS	CfoI	TCCTTTATACCGACTTGAAGCTATTG	AGATTCTATGTTTCTTGAAAGCACAGC
66.	C2At3g44890	11	102.7	CAPS	SpeI	ATTGGGCAAAGCTCAAATTGTGAC	AGCCTCAATTTTCTCGTCTTCCATC
67.	C2At5g60540	11	106.2	PCR	NA	TGCTGTTTTCATCCGTGCTCC	AGTTAATTCGGGATGAAAAGCAG
68.	C2At5g11550	11	108.2	CAPS	MspI	ATTGCCCCTCCTGTTTTGTACAC	CACCGGATTCGGAACAAGTGAATG
69.	C2At2g28250	11	110.2	CAPS	HinfI	AGACTTCATCATCGTCATGTGGTTCCG	TTTGGAGGTGCTTTGCCATACCAAG
70.	C2At4g22260	12	0	CAPS	ScrFI	TCCTCTAACGGTCTAGAGAAATGGG	AGGAACTCTTGCAATTGTTTCCAGAAC
71.	C2At1g79260	12	1.7	dCAPS	CfoI	CCATCATGTTATCAGATTTTTAGATGC	CTTCATGAATGCACCCATAAAATAAG
72.	C2At3g52640	12	3.3	CAPS	CfoI	TACCTTGGCAGTAGAAGATTTCTTCTTG	AACCCTTTCCAACTGATCCAATTTC
73.	C2At5g16630	12	7.5	CAPS	SspI	TAAATGCAATCACTGATGGAGAGCA	TGCCAATACTGCATCCCACCAAAT
74.	C2At5g16710	12	12.7	PCR	NA	TGATGAGCTGACAGCTTTCAATGAT	AGTGAATCTGGAATAGACCAATTCTTAT
75.	T0408	12	17.9	CAPS	NsiI	GCTGCTGGACTCACAGTTGA	TTCTCGGCACCCATTCTAAC
76.	C2At3g60830	12	27.4	CAPS	DpnII	ATGCTGGTTCTAAATTTCTCAAAGC	ATATGCGTCCAACTGCATAAAGCG
77.	C2At3g54840	12	28.9	dCAPS	SspI	CATGTTCTTATATCATGTAACGTTTAA	TGTGCTTTGGCAAAAGACTCAGGAC
78.	C2At2g28600	12	36.4	dCAPS	HinfI	AGCGATGATTCCATTCAGAGAAGG	TTCACGACAATTATTTTCTTTATTGA
79.	C2At4g15010	12	40.8	CAPS	DpnII	ACATCCCAATTTGGTTACTGCCCTG	AGGGGACAATGGACCAACTTCTTCATC
80	C2At5g64730	12	45.6	PCR	NA	GATCGACAAGTATTTTATTGGGATGT	GTAGTCGTCGTTGATTGAGGCATAAT
81.	C2At5g09880	12	50.4	CAPS	MseI	AAAACATGTTTGATCCTGCAACTGAG	CCTTTGAACTTGGCATCATATTCAT
82.	C2At3g24490	12	52.4	CAPS	Hpy188I	AGGAGAAGATGAAGTTTGCAGAGACTG	ATTCTTGCAATTTCTGCCTGAGC
83.	C2At4g39660	12	56.5	CAPS	SspI	ACAGGGAGCCACTACTGGGGTTT	ACATAACCAACAAATAAGGTGCACG
84.	C2At4g39870	12	56.5	CAPS	TaqI	AGACTTAACCAATGCTTCTGTTGGC	AACTAGCCCACCAAACACAGCACC
85.	C2At1g79790	12	57.3	CAPS	ApoI	ATATTCCTACCTTGAAGGTGTTGAAG	AGAGTTTTAGCTCGTCCTCAATCATC
86.	C2At1g65230	12	57.3	dCAPS	HindIII	TTACAGGACGAGACAAGTACAAGAGACC	TTATAATTTGAAAACAGGGTAAAAAGC

* *Source: Wu et al., 2009*.

### Analysis of polymorphism between resistant and susceptible parents and, F_2_ population

Chilli leaf curl virus (ChLCV) susceptible genotypes, Phule Mukta (PM) and Anugraha, as well as resistant genotypes, DLS-Sel-10 and WBC-Sel 5, of hot pepper were used to study the polymorphism in selected markers. Genomic DNA was extracted from young leaf tissue following CTAB method (Murray and Thompson, 1980). DNA quality and quantity were assessed on a 1% (w/v) agarose gel stained with ethidium bromide (Sigma Aldrich Chemical Pvt. Ltd, Bangalore, India) and by using a NanoDrop®ND-1000 spectrophotometer, respectively.

A total of 86 orthologous CAPS/dCAPS/PCR markers between tomato and pepper were selected for the polymorphism analysis (Table 2). Primers were custom synthesized (SBS Genetech Co. Ltd., Beijing, China). All the markers were amplified by PCR in 15 μl reaction volumes with 50 ng genomic DNA, 1.0 μM of each primer and 1.0 unit of *Taq* DNA polymerase (Hi media Laboratories, Mumbai, India). Amplification conditions involved: initial incubation at 94°C for 3 min followed by 30 cycles of 94°C for 0.5 min, 55–65°C (depending on annealing temperature of primers) for 1 min, and 72°C for 1 min; and a final incubation at 72°C for 5 min. Amplified products in case of CAPS and dCAPS markers were digested using specific restriction enzymes as given in the Table 2 as per manufacturer's instructions (Thermo Fisher Scientific Inc.). Amplified and digested products were resolved on 3.0% agarose gels with Tris/Acetate/EDTA (TAE), at a constant voltage of 60 V for 3 h using a horizontal gel electrophoresis system (BioRad, USA). The gels were visualized and photographed under UV light in a gel documentation unit (Alpha imager, Cell Biosciences, Santa Clara, CA).

The polymorphic markers between the parents were used for BSA in F_2_ population derived from crosses of PM X DLS-Sel-10 as well as Anugraha X WBC-Sel 5. Screening for ChLCV was done and symptom severity was scored on individual plants according to scale developed by Kumar et al. (2006) ranging from 0 to 5 with “0” indicating no symptoms and “5” indicating extreme susceptibility with more than 75% curling, deformed small leaves, stunted plant growth with small flowers and no or little fruit set. F_2_ plants showing extreme phenotypes of resistance and susceptibility under challenge inoculation were used for BSA. Ten F_2_ individuals showing resistant phenotype with a score of 0 and ten F_2_ individuals found susceptible with a score of 5 were separately used for the development of bulks. Equal quantities of DNA were bulked from susceptible individuals and resistant individuals to generate two DNA bulks, namely resistant bulk (RB) and susceptible bulk (SB). The susceptible and resistant bulks along with parents were screened with selected markers as described above.

### *In Silico* analysis of conservation/dispersion of genes located within *Ty* QTL regions

In order to determine the physical location of genes present on selected *Ty* QTL regions and their synteny with hot pepper, following strategy was adopted:

First of all, the location of all the markers flanking the QTL of interest was checked on respective chromosomes in tomato EXPEN 2000 map in the Sol Genomics Network (http://www.sgn.cornell.edu) and the physical coordinates of these markers were recorded. Thereafter, the information regarding all the gene models between these coordinates was extracted from International Tomato Genome Sequencing project version 2.4 (https://solgenomics.net/organism/Solanum_lycopersicum/genome). The information regarding syntenic genes in hot pepper for all the tomato genes as well as the physical location of each gene on respective chromosome was downloaded from FTP site at pepper genome database (Pepper Institute, Zunyi Academy of Agricultural Sciences; http://peppersequence.genomics.cn/page/species/index.jsp). The information for selected genes was extracted from source file using Microsoft excel. Gene annotations for tomato and pepper genes were downloaded from Phytozome (https://phytozome.jgi.doe.gov/pz/portal.html) and the pepper genome database, respectively (Tables S6–S10).

## Results

### Orthologous markers between pepper and *Ty* QTL regions of tomato

The detailed results obtained for orthologous markers in all five QTL regions are described below:

#### Orthologous markers on *Ty1* and *Ty3* QTL regions

Ji et al. (2007a) had mapped the begomovirus resistance locus *Ty3* from *S. chilense* on chromosome 6 of tomato near TYLCV resistance locus *Ty1*. *Ty1* locus was mapped to pericentromeric region, whereas, *Ty3* to long arm of chromosome 6. The position of *Ty3* locus in the map was shown between markers T0774 and T1079 and that of *Ty1* locus between markers C2_At4g01900 and C2_At3g10920 (Table 1). We selected all the orthologous markers between C2_At4g01900 and T1079 so that all the markers located in the region harboring both *Ty1* and *Ty3* QTLs could be utilized to study the polymorphism in ChLCV resistant and susceptible pepper lines. The *Ty1* flanking marker C2_At4g01900 was found on pepper chromosome 6 at 0 cM position, whereas, orthologous markers C2_At3g56130 and C2-At1g06110 flanked *Ty3* locus on pepper chromosome 6. Therefore, all the markers between C2_At4g01900 (0 cM) and C2-At1g06110 (55.9c M) as well as few additional markers up to 110.3 cM in pepper CosII map of chromosome 6 were shortlisted for studying the polymorphism. In total, 17 orthologous CAPS markers were selected from this region (Table 2).

#### Orthologous markers on *Ty2* QTL region

*Ty2* QTL initially identified from *S. habrochaites* (B6013) was mapped to chromosome 11 in the 19 cM region on the long arm flanked by markers TG36 and cLET-24-J2A (Hanson et al., 2006; Ji et al., 2007b, 2009a). On comparison of Tomato EXPEN 2000 and pepper COSII maps, it was observed that markers between TG36 and CLET-24-J2A (85–95 cM) on chromosome 11 of tomato have dispersed in such a way that TG 36 which is at 84 cM on chromosome 11 of tomato occupied position at 115.5 cM on chromosome 11 of pepper. Whereas, orthologs of some of the markers located between 85 and 95 cM region in tomato mapped between 100 and 110 cM region in pepper. Some of the markers orthologous to chromosome 11 of tomato also mapped to chromosome 12 of pepper. We included all the orthologous markers located between 91.9 and 110.2 cM on pepper chromosome 11 and between 0 and 57.3 cM region on chromosome 12 of pepper of COSII map. In total, 23 orthologous CAPS markers were selected from this region (Table 2).

#### Orthologous markers on *Ty4* QTL region

The *Ty4* locus was mapped to chromosome 3 of tomato in some resistant breeding lines derived from LA1932 of *S. Chilense* in the 2.3 cM interval between C2_At4g17300 and C2_At5g60160. Although these markers were not found on COS II map, markers C2_At5g62390 and C2_At5g52820, which flank *Ty4* locus on both sides (Ji et al., 2009b) could be located at 63.3 and 109.5 cM, respectively on the long arm of chromosome 3 of pepper. We selected all the 22 orthologous markers located between 41.5 and 128 cM region of chromosome 3 of pepper for the current study (Table 2).

#### Orthologous markers on *ty5* QTL region

Tomato yellow leaf curl virus (TYLCV) resistance gene *ty5* has been reported to account for 39.7–46.6% of the variation in symptom severity among segregating plants. Anbinder and coworkers (2009) mapped this gene to chromosome 4 near the marker SlNAC1 which was at 13.5 cM from marker J04-1 and at 17.1 cM from TG182. Later, Kadirvel and co-workers (2013) reported that SSR43 flank SINAC1 on one side. We tried to locate these markers on chromosome 4 of tomato and compared it with the map of chromosome 4 of pepper in COSII. However, there were no orthologous markers in this region. Whereas, when chromosome 4 of Tomato EXPEN 2000 map was compared with chromosome 5 of pepper COSII map, some orthologous markers could be identified in this region. Therefore, we selected orthologous markers located between 139.6 and 158.6 cM on chromosome 4 (6 markers) as well as between 0 and 82.1 cM on chromosome 5 (18 markers) of pepper for our study (Table 2).

### Survey of polymorphism between resistant and susceptible parents and, F_2_ population

A total of 86 orthologous markers were evaluated in four pepper genotypes: the susceptible genotypes, PM and Anugraha, as well as resistant genotypes, DLS-Sel-10 and WBC-Sel -5. Four markers, namely C2At5g11550 (located on chromosome 11 and selected for testing *Ty2* QTL synteny), C2At5g23060 (located on chromosome 3 and selected for *Ty4* QTL synteny), C2At3g55800 (located on chromosome 5 and selected for *ty5* QTL synteny) and C2At5g17170 (located on chromosome 3 and selected for *Ty4* QTL synteny) were found to be polymorphic between the resistant and susceptible parents (Figure S1). These markers were then tested using BSA on F_2_ population derived from the cross between PM X DLS-Sel-10 and Anugraha X WBC-Sel. None of the markers showed polymorphism in BSA thereby, indicating lack of linkage between the tested markers and resistance trait.

### Analysis of synteny and micro-collinearity between pepper and tomato genomes at *Ty* QTL regions

**Figure 1:**
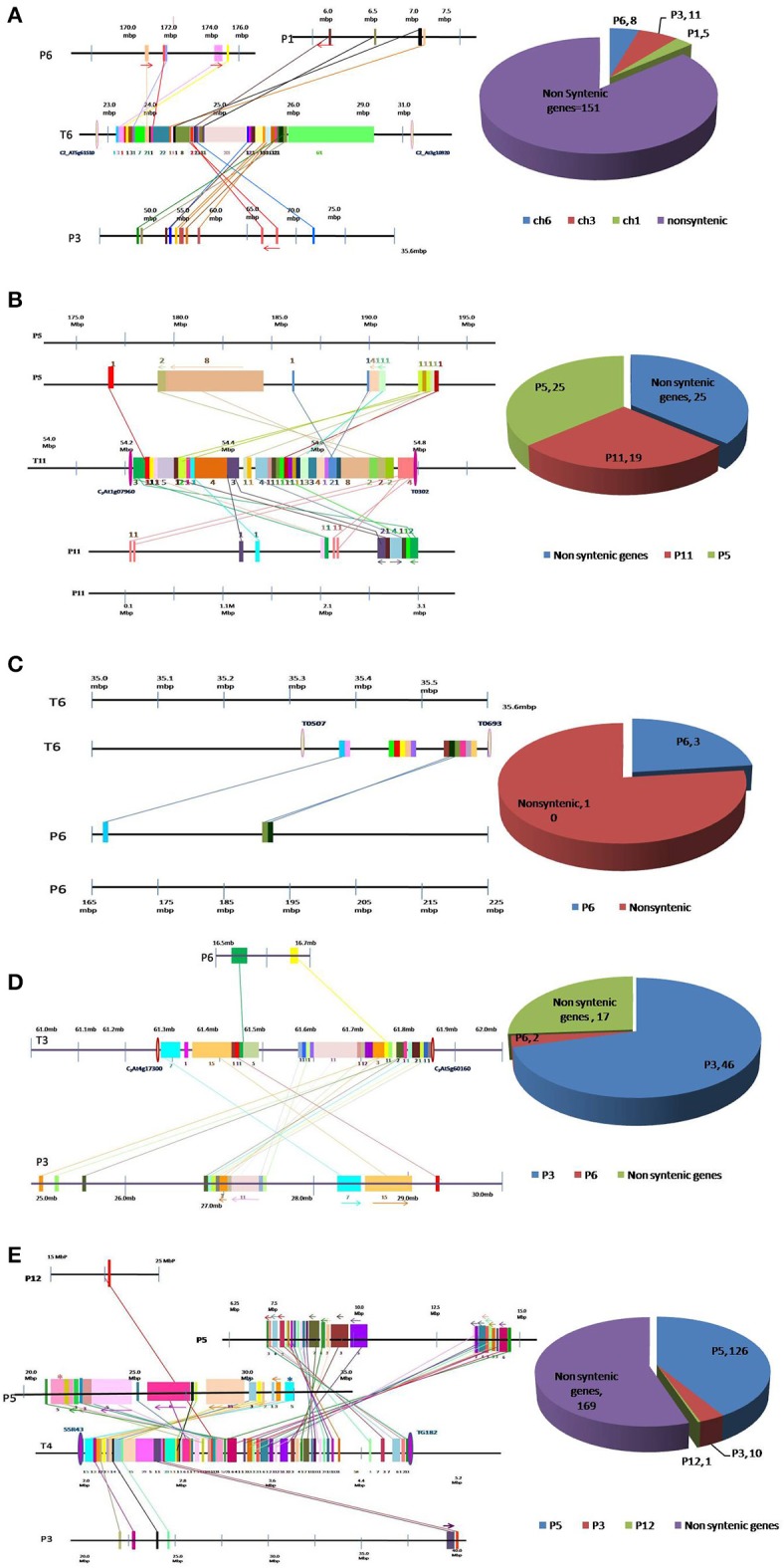
Conservation and dispersion of genes located in **(A)** *Ty1*, **(B)** *Ty2*, **(C)** *Ty3*, **(D)** *Ty4*, and **(E)** *ty5* QTL regions of tomato and orthologous regions from pepper. The region on the left represents synteny and micro-collinearity between tomato *Ty* QTL region and orthologous regions in pepper. The chromosomal regions are represented by horizontal lines, whereas, vertical bars represent genes. The tomato chromosome number is indicated with “T” followed by number, whereas pepper chromosome number is indicated by “P” followed by number. Orthologous genes are shown by same color bar and are joined through a line. The arrows indicate change in orientation of genes in pepper genome. The region on the right presents distribution of syntenic and non-syntenic orthologs identified in pepper. Numbers of non-syntenic genes and syntenic genes found on each chromosome are given.

Since, we did not observe the linkage in polymorphic markers tested in this study, we further examined the syntenic relationship and order of the genes in the selected QTL regions between tomato and pepper, so as to unravel the pattern of conservation/dispersion of homologous segments within these QTL regions. The markers flanking *Ty* QTLs in tomato and their physical location on respective chromosomes is shown in Table 1. The details of tomato genes located within each QTL region, their orthologs and physical location have been provided in Tables S1–S5. The pattern of conservation and dispersion of genes located within each QTL region between tomato and pepper has been displayed in Figures 1A–E.

Out of 175 tomato genes located within *Ty1* QTL region, only 24 were found to be conserved in pepper genome (Figure 1A). Moreover, these 24 genes were dispersed in three pepper chromosomes *viz*. chromosome 1 (5 genes), chromosome 3 (11 genes) and chromosome 6 (8 genes). Clearly, orthologous genes in this QTL region were not located on big syntenic blocks but instead seem to have dispersed to different chromosomes of pepper genome (Figure 1A; Table S1).

Tomato chromosome 11 harbors 69 genes within *Ty2* QTL region, out of which 44 were found to be conserved in the pepper genome (Figure 1B). Among these, 19 were located on chromosome 11, whereas, 25 genes were found on chromosome 5 of pepper. Though this region exhibited longer syntenic regions, the order of genes was not always same as in tomato genome (Figure 1B; Table S2). For example, a block of eight tomato genes comprising Solyc11g070100.1, Solyc11g070110.1, Solyc11g070120.1, Solyc11g070130.1, Solyc11g070140.1, Solyc11g070150.1, Solyc11g070160.1, and Solyc11g070170.1 was located between 56.63 and 54.69 Mb region of tomato chromosome 11. The orthologous genes to this region were identified on a single chromosome (chromosome 5) in pepper but the order of genes had reversed (between 179.74 and 184.53 Mb).

In LA1932-derived advanced lines, *Ty3* locus has been mapped within a short distance between markers T0507 and T0693 on chromosome 6 (Ji et al., 2007a). Thirteen genes were located between these markers in tomato. However, orthologs of only three of them could be located in pepper genome (Figure 1C, Table S3).

Sixty-five genes were found in a 0.54 Mb region (from 61.28 to 61.82 Mb) harboring *Ty4* QTL on tomato chromosome 3. Orthologs for 48 of them were identified in pepper genome (2 on chromosome 6 and 46 on chromosome 3; Figure 1D) Among these, 46 genes were dispersed in 4.15 Mb region on pepper chromosome 3 (from 25.19 to 29.34 Mb). Syntenic blocks in this region comprised 7–15 genes, however, gene order was altered in most of the segments (indicated by forward and backward arrows in Figure 1D). Overall, in spite of high level of synteny, gross changes in position of genes were observed in this region of pepper and tomato genomes (Table S4).

Due to non-availability of closely-linked markers, a 2.7 Mb region (from 2.13 to 4.83 Mb) of tomato chromosome 4 harboring *ty5* QTL was used to study the synteny between tomato and pepper genomes in this region. A total of 306 genes were found in this region in tomato genome, however orthologs of only 137 genes could be identified in pepper genome (Figure 1E; Table S4). Among these, 126 genes were found on pepper chromosome 5, concentrated in two regions, one region from 7.58 to 14.68 Mb harboring 68 genes and another region from 21.46 to 32.03 Mb harboring 58 genes. The gene order in different segments which had dispersed to either different chromosomes or different regions of the same chromosome was conserved in some cases, reversed in others and reshuffled in a few cases.

## Discussion

Whitefly-transmitted begomoviruses are responsible for heavy losses in the yields of several vegetable and staple food crops, worldwide. With the changing climatic conditions and agricultural practices, the prevalence and distribution of these viruses has significantly increased causing a global concern. TYLCV, a monopartite virus, is one of the examples from this category where novel strains have been reported from diverse hosts and habitats, not detected earlier (Pratap et al., 2011; Fortes et al., 2016). Considerable work has been carried out in identification and utilization of QTLs/genes responsible for conferring resistance to the TYLCV in tomato. Leaf curl begomoviruses are known to infect a range of host species within family Solanaceae and often mixed infection with multiple viruses have been reported which lead to increase in severity of symptoms (Srivastava et al., 2015; Singh et al., 2016). Although, the role of *Ty* genes in conferring resistance to ChLCV, another monopartite virus (George et al., 2014) remains to be tested, recent studies show that *Ty* genes can confer resistance to both monopartite and bipartite begomoviruses (Prasanna et al., 2015; Fortes et al., 2016).

Structural and functional conservation in R genes has been reported among several related hosts species (Yu et al., 1996; Dijan-Caporalino et al., 1998; Grube et al., 2000). Several studies in the past, using common genetic markers, revealed conservation of large tracts of collinear markers in solanaceous genomes (Bonierbale et al., 1988; Tanksley et al., 1992; Doganlar et al., 2002). Since, there is high level of synteny and collinearity between tomato and pepper genomes, analysis of structural and functional conservation of *Ty* QTL regions in tomato and pepper genomes has promising implications in deciphering possible role of *Ty* orthologs in conferring resistance to ChCLV in pepper (Grube et al., 2000).

Pepper (*C. annuum* L.) is one of the few plant species in which pioneering work of comparative genetic mapping using DNA-based markers was done (Tanksley et al., 1988; Prince et al., 1993). Thereafter, several genetic maps were developed in other solanaceous crops based on different DNA marker systems, further improving the genome coverage, marker density and insights into synteny (Livingstone et al., 1999; Kang et al., 2001; Lefebvre et al., 2002; Lee et al., 2004; Paran et al., 2004; Ben-Chaim et al., 2006; Minamiyama et al., 2006). Wu et al. (2009) developed a linkage map of pepper comprising 299 conserved orthologous markers (COSII) and inferred the probable positions of additional 288 COSII markers utilizing synteny between pepper and tomato genomes. Overall, 587 orthologous markers were reported by them in pepper genome. Based on the results, 35 conserved syntenic segments with well-preserved order of genes were reported in pepper and tomato genomes (Wu et al., 2009). In this study, we leveraged this resource to identify the orthologous markers in pepper corresponding to *Ty* QTL regions of tomato.

We shortlisted 86 orthologous markers, corresponding to different Ty loci of tomato, in pepper and tested them for polymorphism in ChCLV-susceptible and resistant genotypes of pepper. Four markers, polymorphic between the resistant and susceptible parents, were subsequently used for BSA in F_2_ population. The fundamental principle of BSA is that if there is a molecular marker that shows polymorphism between the parents of a population and is closely linked to major QTL/gene controlling a particular trait, it should co-segregate with the QTL (Quarrie et al., 1999). Therefore, two DNA pools developed from F_2_ plants showing contrasting trait (extreme resistance and extreme susceptibility to ChLCV) were evaluated to identify polymorphic markers between them so as to confirm the linkage of the marker to the loci determining the trait. However, since in the current study, none of the markers showed polymorphism in BSA, it indicates that these polymorphic markers were not segregating with the trait controlling resistance/susceptibility to ChLCV and, therefore, are not linked to the trait of our interest. This can be explained by gross rearrangements in plant genome during evolution. The decay of R-gene collinearity among plant species has been especially attributed to tandem and segmental duplications that eventually lead to copy number and presence-absence variations (Zhang et al., 2014).

To further examine the level of synteny and micro-collinearity among genes lying in *Ty* QTLs of tomato and the orthologous regions in pepper, we leveraged the information available for syntenic regions in tomato and pepper genomes at pepper genome database. The concept of synteny which pertains to the “preserved co-location of homologous genes on chromosomes between species, irrespective of genetic linkage and gene order” was introduced in 1971 (Ehrlich et al., 1997; Passarge et al., 1999; Peters et al., 2012). Organization of genome, diversification of genes and evolutionary relationships between various solanaceous crops has been investigated by different workers using synteny and conserved linkage (Ku et al., 2000; Fulton, 2002; Wu et al., 2006; Wang et al., 2008). Conservation in the order and sequence of orthologs was reported in most of these cases, barring a few small-scale differences and positive gene selections (Doganlar et al., 2002; Wang et al., 2008). Two recent publications also suggested strong collinearity of the pepper genome with that of tomato (Kim et al., 2014; Qin, 2014).

In the present study, we observed that 13.71% genes located in the *Ty1* QTL region, 63.76% in *Ty2* QTL region, 23.07% in *Ty3* QTL region, 73.84% located in *Ty4* QTL region and 44.77% of genes located in *ty5* QTL region in tomato were found to be conserved in pepper genome. However, despite such high conservation in some QTLs, the linkage relationship between different genes was greatly affected due to gross rearrangements with respect to order and position of genes in these species. The results appear to be in agreement with the earlier reports showing that the linear order of the genes in tomato and pepper chromosomes has been greatly modified due to rearrangements (Tanksley et al., 1988; Prince et al., 1993; Livingstone et al., 1999). Chromosomal rearrangements within solanaceous genomes (5 between potato and tomato and 30 rearrangements between pepper and tomato) have been reported in several other studies as well (Bonierbale et al., 1988; Tanksley et al., 1988; Prince et al., 1993; Livingstone et al., 1999; Wang et al., 2008). Significant rearrangements involving inversions and segmental translocations have been reported in the euchromatin regions of tomato, potato and pepper (Peters et al., 2012). We also noticed several gene rearrangements both within and between chromosomes as well as local gene rearrangements between tomato and pepper genomes in the *Ty* QTL regions investigated in this study. A closer look at the annotations of orthologous *Ty* regions in tomato and pepper genomes (Tables S6–S10) revealed that a large number of genes coding for proteins belonging to the nucleotide binding site-leucine-rich repeat (*NBS-LRR*) family present in tomato *Ty* QTLs are not conserved in pepper which might be partially responsible for the lack of linkage between the markers used in the study. Conversely, several disease resistance-related genes and transcription factors in the selected QTLs were found to be conserved in both the genomes. Further, functional characterization of these genes has potential to provide a way forward for begomovirus resistance breeding in pepper.

## Conclusion

The present study provides useful information regarding conservation and dispersion of genes located within five quantitative trait loci conferring resistance to TYLCV of tomato between pepper and tomato genomes. The study revealed significant synteny in *Ty* QTLs in both the genomes. The micro-collinearity however, seems to be greatly affected by genomic rearrangements including inversions, deletions and reshuffling of gene order. These rearrangements are largely responsible for the lack of linkage relationship between orthologous markers in these species. The study provides an important insight into structural changes that may lead to variability in disease resistance in related plant species.

## Author contributions

MM performed the experiments, carried out *in silico* analysis and wrote the MS. AS developed mapping populations and looked after the breeding aspect of study. RS helped in the bioinformatics analysis and revision of the manuscript. PK supervised the work and contributed in the revision of manuscript.

### Conflict of interest statement

The authors declare that the research was conducted in the absence of any commercial or financial relationships that could be construed as a potential conflict of interest.
