# Supplementary material for: Conservation and Dispersion of Genes Conferring Resistance to Tomato Begomoviruses between Tomato and Pepper Genomes
Source: Front Plant Sci. 2017 Nov 7;8:1803. doi: 10.3389/fpls.2017.01803 (PMC5681951; doi:10.3389/fpls.2017.01803)
Supplement: Supplementary file 1 [file Table1.PDF]

**Table S1. Genes located within *Ty1* QTL region of tomato and their orthologs in pepper**

| S.No | Genes in Tomato | Location in Tomato | | Orthologs in Pepper | Location in Pepper | |
|---|---|---|---|---|---|---|
| | | | | | | |
| 1. | Solyc06g034130.2 | 23703696 | 23705148 | | | |
| 2. | Solyc06g034140.1 | 23707557 | 23709701 | capana06g002534 | 174553523 | 174565323 |
| 3. | Solyc06g034150.2 | 23710658 | 23717769 | capana06g002538 | 175129304 | 175132311 |
| 4. | Solyc06g034160.2 | 23724551 | 23733517 | capana06g002539 | 175279253 | 175282171 |
| 5. | Solyc06g034170.1 | 23771302 | 23771628 | | | |
| 6. | Solyc06g034180.1 | 23801802 | 23804815 | capana06g002547 | 175430396 | 175437797 |
| 7. | Solyc06g034190.1 | 23817722 | 23818406 | | | |
| 8. | Solyc06g034200.1 | 23829955 | 23831559 | | | |
| 9. | Solyc06g034210.1 | 23945509 | 23945757 | | | |
| 10. | Solyc06g034220.2 | 23958601 | 23964557 | capana06g002527 | 172021681 | 172022382 |
| 11. | Solyc06g034230.1 | 23967209 | 23969451 | | | |
| 12. | Solyc06g034250.1 | 23987782 | 23989423 | | | |
| 13. | Solyc06g034260.1 | 23997949 | 23998882 | | | |
| 14. | Solyc06g034290.2 | 24019882 | 24021762 | | | |
| 15. | Solyc06g034300.1 | 24029545 | 24029715 | | | |
| 16. | Solyc06g034310.2 | 24031634 | 24033824 | | | |
| 17. | Solyc06g034320.1 | 24034960 | 24035133 | | | |
| 18. | Solyc06g034330.2 | 24061458 | 24079233 | capana06g002522 | 171290124 | 171292878 |
| 19. | Solyc06g034340.1 | 24084919 | 24086861 | capana06g002524 | 171374983 | 171383711 |
| 20. | Solyc06g034350.1 | 24131946 | 24133138 | | | |
| 21. | Solyc06g034360.1 | 24172403 | 24176069 | capana06g002526 | 172020164 | 172020595 |
| 22. | Solyc06g034370.1 | 24179516 | 24180094 | | | |
| 23. | Solyc06g034380.1 | 24201838 | 24202312 | | | |
| 24. | Solyc06g034390.1 | 24216543 | 24217583 | | | |
| 25. | Solyc06g034400.1 | 24228884 | 24229072 | | | |
| 26. | Solyc06g034410.2 | 24265510 | 24266336 | | | |
| 27. | Solyc06g035410.1 | 24292825 | 24293946 | | | |
| 28. | Solyc06g035420.1 | 24304430 | 24306406 | | | |
| 29. | Solyc06g035430.2 | 24308994 | 24313705 | | | |
| 30. | Solyc06g035440.2 | 24342167 | 24346731 | | | |
| 31. | Solyc06g035450.2 | 24350869 | 24354981 | | | |
| 32. | Solyc06g035460.2 | 24354411 | 24356915 | | | |
| 33. | Solyc06g035470.1 | 24358915 | 24360639 | | | |
| 34. | Solyc06g035480.2 | 24361306 | 24363602 | | | |
| 35. | Solyc06g035490.2 | 24377395 | 24379035 | | | |
| 36. | Solyc06g035500.1 | 24420192 | 24420367 | | | |
| 37. | Solyc06g035510.1 | 24422188 | 24424559 | | | |
| 38. | Solyc06g035520.2 | 24439422 | 24441001 | | | |
| 39. | Solyc06g035530.2 | 24482418 | 24484616 | | | |
| 40. | Solyc06g035540.1 | 24502743 | 24502901 | | | |
| 41. | Solyc06g035550.2 | 24522827 | 24523003 | | | |

| | | | | | | |
|---|---|---|---|---|---|---|
| 42. | Solyc06g035560.1 | 24569808 | 24570132 | | | |
| 43. | Solyc06g035570.1 | 24580576 | 24580740 | | | |
| 44. | Solyc06g035580.2 | 24597492 | 24603412 | capana01g000403 | 7366130 | 7366660 |
| 45. | Solyc06g035590.1 | 24652232 | 24653360 | | | |
| 46. | Solyc06g035600.2 | 24661966 | 24664957 | capana01g000397 | 7170442 | 7171112 |
| 47. | Solyc06g035610.2 | 24665930 | 24667163 | | | |
| 48. | Solyc06g035620.2 | 24667330 | 24668828 | | | |
| 49. | Solyc06g035630.1 | 24668918 | 24669235 | | | |
| 50. | Solyc06g035640.1 | 24671390 | 24671782 | | | |
| 51. | Solyc06g035650.1 | 24694401 | 24694733 | | | |
| 52. | Solyc06g035660.1 | 24701688 | 24703669 | | | |
| 53. | Solyc06g035670.1 | 24704389 | 24705322 | | | |
| 54. | Solyc06g035680.1 | 24707439 | 24708331 | | | |
| 55. | Solyc06g035690.2 | 24713848 | 24720021 | capana03g002393 | 65524820 | 65526144 |
| 56. | Solyc06g035700.1 | 24738494 | 24739030 | capana03g002388 | 65249521 | 65250168 |
| 57. | Solyc06g035710.1 | 24772352 | 24773914 | capana01g000327 | 6172759 | 6173394 |
| 58. | Solyc06g035720.2 | 24816413 | 24819781 | capana01g000326 | 6159030 | 6161280 |
| 59. | Solyc06g035740.1 | 24872518 | 24876166 | capana03g002469 | 73817663 | 73821080 |
| 60. | Solyc06g035750.1 | 24888868 | 24889167 | | | |
| 61. | Solyc06g035760.2 | 24907105 | 24908892 | capana01g000367 | 6767159 | 6768855 |
| 62. | Solyc06g035770.1 | 24938877 | 24939432 | | | |
| 63. | Solyc06g035790.2 | 25033752 | 25037135 | | | |
| 64. | Solyc06g035800.1 | 25041694 | 25042059 | | | |
| 65. | Solyc06g035810.1 | 25054917 | 25055473 | | | |
| 66. | Solyc06g035820.1 | 25056565 | 25056864 | | | |
| 67. | Solyc06g035830.1 | 25084660 | 25086052 | | | |
| 68. | Solyc06g035840.1 | 25105456 | 25105674 | | | |
| 69. | Solyc06g035850.1 | 25105840 | 25106136 | | | |
| 70. | Solyc06g035860.1 | 25112829 | 25113178 | | | |
| 71. | Solyc06g035870.2 | 25115574 | 25117598 | | | |
| 72. | Solyc06g035880.1 | 25159418 | 25159946 | | | |
| 73. | Solyc06g035890.1 | 25194211 | 25194456 | | | |
| 74. | Solyc06g035900.1 | 25233598 | 25233861 | | | |
| 75. | Solyc06g035910.1 | 25254826 | 25255044 | | | |
| 76. | Solyc06g035920.2 | 25270356 | 25277247 | | | |
| 77. | Solyc06g035930.1 | 25281564 | 25283374 | | | |
| 78. | Solyc06g035940.2 | 25290922 | 25306791 | | | |
| 79. | Solyc06g035960.2 | 25322428 | 25326723 | | | |
| 80. | Solyc06g035970.2 | 25326805 | 25329776 | | | |
| 81. | Solyc06g035980.2 | 25341865 | 25342802 | | | |
| 82. | Solyc06g035990.2 | 25351566 | 25353875 | | | |
| 83. | Solyc06g036000.2 | 25362564 | 25373975 | | | |
| 84. | Solyc06g036010.1 | 25403187 | 25405805 | | | |
| 85. | Solyc06g036020.1 | 25407734 | 25409718 | | | |

| | | | | | | |
|---|---|---|---|---|---|---|
| 86. | Solyc06g036030.1 | 25424468 | 25425166 | | | |
| 87. | Solyc06g036040.1 | 25427289 | 25428953 | | | |
| 88. | Solyc06g036050.2 | 25429691 | 25431549 | | | |
| 89. | Solyc06g036060.2 | 25438412 | 25446863 | | | |
| 90. | Solyc06g036070.2 | 25449729 | 25457547 | | | |
| 91. | Solyc06g036080.2 | 25466708 | 25477184 | | | |
| 92. | Solyc06g036090.1 | 25490979 | 25491541 | | | |
| 93. | Solyc06g036100.2 | 25521665 | 25524159 | | | |
| 94. | Solyc06g036110.1 | 25557152 | 25557898 | | | |
| 95. | Solyc06g036120.2 | 25573431 | 25575004 | | | |
| 96. | Solyc06g036130.2 | 25575619 | 25579622 | capana03g002183 | 52361164 | 52367180 |
| 97. | Solyc06g036140.1 | 25585970 | 25586491 | | | |
| 98. | Solyc06g036150.1 | 25601649 | 25607510 | | | |
| 99. | Solyc06g036170.1 | 25637986 | 25639782 | capana03g002179 | 52120094 | 52122085 |
| 100. | Solyc06g036180.2 | 25663317 | 25664078 | | | |
| 101. | Solyc06g036190.1 | 25665582 | 25665782 | | | |
| 102. | Solyc06g036200.1 | 25684352 | 25685771 | | | |
| 103. | Solyc06g036210.1 | 25686378 | 25687285 | | | |
| 104. | Solyc06g036220.1 | 25688216 | 25690397 | capana03g002234 | 54730461 | 54736285 |
| 105. | Solyc06g036230.2 | 25707674 | 25708131 | | | |
| 106. | Solyc06g036240.1 | 25709842 | 25716466 | | | |
| 107. | Solyc06g036250.1 | 25730669 | 25731298 | | | |
| 108. | Solyc06g036260.2 | 25742578 | 25744513 | capana03g002254 | 55520262 | 55523330 |
| 109. | Solyc06g036270.1 | 25762284 | 25765061 | capana03g002236 | 54790658 | 54791020 |
| 110. | Solyc06g036290.2 | 25803161 | 25806396 | capana03g002239 | 54805795 | 54808789 |
| 111. | Solyc06g036300.2 | 25807174 | 25815388 | | | |
| 112. | Solyc06g036310.2 | 25865849 | 25866721 | capana03g002240 | 54856742 | 54858861 |
| 113. | Solyc06g036320.1 | 25884175 | 25885400 | | | |
| 114. | Solyc06g036330.1 | 25902106 | 25903086 | | | |
| 115. | Solyc06g036340.2 | 25903516 | 25908919 | capana03g002162 | 49832880 | 49839819 |
| 116. | Solyc06g036350.2 | 25916880 | 25922222 | | | |
| 117. | Solyc06g036360.1 | 25926320 | 25926857 | | | |
| 118. | Solyc06g036370.1 | 25929032 | 25929625 | | | |
| 119. | Solyc06g036380.1 | 25930365 | 25934152 | | | |
| 120. | Solyc06g036390.1 | 25950082 | 25950372 | | | |
| 121. | Solyc06g036410.1 | 25974062 | 25974262 | | | |
| 122. | Solyc06g036420.1 | 25974441 | 25977666 | | | |
| 123. | Solyc06g036430.2 | 25980533 | 25983217 | | | |
| 124. | Solyc06g036440.1 | 25990942 | 25993563 | | | |
| 125. | Solyc06g036450.1 | 26001329 | 26001631 | | | |

| | | | | | | |
|---|---|---|---|---|---|---|
| 126. | Solyc06g036460.2 | 26022912 | 26023938 | | | |
| 127. | Solyc06g036470.1 | 26099905 | 26103421 | | | |
| 128. | Solyc06g036480.1 | 26104474 | 26104982 | | | |
| 129. | Solyc06g036490.1 | 26105128 | 26109587 | | | |
| 130. | Solyc06g036500.1 | 26145924 | 26146790 | | | |
| 131. | Solyc06g036510.1 | 26426743 | 26428874 | | | |
| 132. | Solyc06g036520.1 | 26433202 | 26433411 | | | |
| 133. | Solyc06g036530.1 | 26442173 | 26443584 | | | |
| 134. | Solyc06g036540.1 | 26448995 | 26449222 | | | |
| 135. | Solyc06g036550.1 | 26473606 | 26473818 | | | |
| 136. | Solyc06g036570.1 | 26668384 | 26668611 | | | |
| 137. | Solyc06g036580.2 | 26685215 | 26688466 | | | |
| 138. | Solyc06g036590.1 | 26697101 | 26697760 | | | |
| 139. | Solyc06g036600.1 | 26761394 | 26764959 | | | |
| 140. | Solyc06g036610.1 | 26842472 | 26842885 | | | |
| 141. | Solyc06g036620.1 | 26964000 | 26969589 | | | |
| 142. | Solyc06g036630.1 | 26972144 | 26973183 | | | |
| 143. | Solyc06g036640.2 | 27000059 | 27005152 | | | |
| 144. | Solyc06g036650.1 | 27009874 | 27010083 | | | |
| 145. | Solyc06g036660.1 | 27140497 | 27140694 | | | |
| 146. | Solyc06g036670.1 | 27145179 | 27146369 | | | |
| 147. | Solyc06g036680.1 | 27161959 | 27163351 | | | |
| 148. | Solyc06g036690.1 | 27172743 | 27176009 | | | |
| 149. | Solyc06g036700.1 | 27178683 | 27180464 | | | |
| 150. | Solyc06g036710.1 | 27199319 | 27202913 | | | |
| 151. | Solyc06g036720.2 | 27265542 | 27282417 | | | |
| 152. | Solyc06g036750.1 | 27489725 | 27492190 | | | |
| 153. | Solyc06g036770.1 | 27640912 | 27641476 | | | |
| 154. | Solyc06g036780.2 | 27821639 | 27838569 | | | |
| 155. | Solyc06g036790.1 | 27846378 | 27846534 | | | |
| 156. | Solyc06g036800.1 | 27890108 | 27890868 | | | |
| 157. | Solyc06g036810.1 | 28060090 | 28061188 | | | |
| 158. | Solyc06g036820.1 | 28232362 | 28232805 | | | |
| 159. | Solyc06g036850.1 | 28271318 | 28271641 | | | |
| 160. | Solyc06g036890.1 | 28289726 | 28290244 | | | |
| 161. | Solyc06g043370.1 | 28612983 | 28618855 | | | |
| 162. | Solyc06g043360.1 | 28628649 | 28628960 | | | |
| 163. | Solyc06g043350.1 | 28643534 | 28643692 | | | |
| 164. | Solyc06g043340.1 | 28643919 | 28644563 | | | |
| 165. | Solyc06g043320.1 | 28706889 | 28707065 | | | |
| 166. | Solyc06g043280.1 | 28861280 | 28861435 | | | |
| 167. | Solyc06g043270.1 | 28878095 | 28878376 | | | |
| 168. | Solyc06g043260.1 | 28889190 | 28889403 | | | |
| 169. | Solyc06g043250.2 | 28915259 | 28919062 | | | |

| | | | | | | |
|---|---|---|---|---|---|---|
| 170. | Solyc06g043240.1 | 29135922 | 29136689 | | | |
| 171. | Solyc06g043230.1 | 29139401 | 29141103 | | | |
| 172. | Solyc06g043210.1 | 29300801 | 29301933 | | | |
| 173. | Solyc06g043180.1 | 29631342 | 29634782 | | | |
| 174. | Solyc06g043170.2 | 29637218 | 29649821 | | | |
| 175. | Solyc06g043160.1 | 29681832 | 29681990 | | | |

**Table S2. Genes located within *Ty*2 QTL region of tomato and their orthologs in pepper**

| S.No. | Genes in Tomato | Location in tomato | | orthologs in pepper | Location in pepper | |
|---|---|---|---|---|---|---|
| | | Start | end | | | |
| 1. | Solyc11g069590.1 | 54221942 | 54224401 | capana11g000063 | 2034036 | 2035670 |
| 2. | Solyc11g069600.1 | 54231913 | 54237114 | capana11g000100 | 2945679 | 2949337 |
| 3. | Solyc11g069610.1 | 54246874 | 54257604 | capana11g000099 | 2934648 | 2937508 |
| 4. | Solyc11g069620.1 | 54262517 | 54266331 | capana05g001827 | 176240167 | 176246353 |
| 5. | Solyc11g069630.1 | 54268154 | 54270222 | | | |
| 6. | Solyc11g069640.1 | 54271866 | 54274489 | capana11g000062 | 2026236 | 2031811 |
| 7. | Solyc11g069650.1 | 54286127 | 54286740 | | | |
| 8. | Solyc11g069660.1 | 54287954 | 54290581 | | | |
| 9. | Solyc11g069670.1 | 54292371 | 54292547 | | | |
| 10. | Solyc11g069680.1 | 54297762 | 54299186 | | | |
| 11. | Solyc11g069690.1 | 54300109 | 54305861 | | | |
| 12. | Solyc11g069700.1 | 54308350 | 54310013 | capana11g000089 | 2817667 | 2821557 |
| 13. | Solyc11g069710.1 | 54310842 | 54319192 | capana05g002016 | 193257483 | 193271962 |
| 14. | Solyc11g069720.1 | 54328528 | 54334085 | capana05g002005 | 192545716 | 192547110 |
| 15. | Solyc11g069730.1 | 54336424 | 54336750 | | | |
| 16. | Solyc11g069740.1 | 54346492 | 54348938 | capana11g000042 | 1357334 | 1359140 |
| 17. | Solyc11g069750.1 | 54356645 | 54358559 | | | |
| 18. | Solyc11g069760.1 | 54376568 | 54377119 | | | |
| 19. | Solyc11g069770.1 | 54398695 | 54403014 | | | |
| 20. | Solyc11g069780.1 | 54405133 | 54410306 | | | |
| 21. | Solyc11g069790.1 | 54418951 | 54423859 | capana11g000039 | 1198410 | 1204720 |
| 22. | Solyc11g069800.1 | 54429028 | 54430560 | capana11g000083 | 2782293 | 2782715 |
| 23. | Solyc11g069810.1 | 54437407 | 54444493 | capana11g000082 | 2769457 | 2773533 |
| 24. | Solyc11g069820.1 | 54450965 | 54460575 | capana05g002017 | 193273712 | 193279658 |
| 25. | Solyc11g069830.1 | 54461801 | 54468555 | | | |
| 26. | Solyc11g069840.1 | 54470088 | 54476360 | capana11g000091 | 2843774 | 2858355 |
| 27. | Solyc11g069850.1 | 54478231 | 54481292 | capana11g000092 | 2866111 | 2867508 |
| 28. | Solyc11g069860.1 | 54483125 | 54487540 | capana11g000093 | 2872568 | 2873746 |
| 29. | Solyc11g069870.1 | 54488988 | 54489530 | capana11g000094 | 2877914 | 2885002 |
| 30. | Solyc11g069880.1 | 54492326 | 54492964 | | | |
| 31. | Solyc11g069890.1 | 54501584 | 54506346 | capana11g000095 | 2893780 | 2899632 |
| 32. | Solyc11g069900.1 | 54519021 | 54519194 | | | |
| 33. | Solyc11g069910.1 | 54521882 | 54524381 | capana11g000096 | 2903842 | 2906155 |

| | | | | | | |
|---|---|---|---|---|---|---|
| 34. | Solyc11g069920.1 | 54526737 | 54527382 | capana05g002018 | 193360554 | 193367028 |
| 35. | Solyc11g069930.1 | 54529077 | 54532865 | | | |
| 36. | Solyc11g069940.1 | 54539246 | 54539674 | capana05g002010 | 192979948 | 192980361 |
| 37. | Solyc11g069950.1 | 54546243 | 54555501 | | | |
| 38. | Solyc11g069960.1 | 54560213 | 54563169 | capana05g001985 | 191291344 | 191294921 |
| 39. | Solyc11g069970.1 | 54565833 | 54566618 | capana05g001984 | 191286061 | 191289136 |
| 40. | Solyc11g069980.1 | 54574400 | 54577231 | capana05g001983 | 191271784 | 191272296 |
| 41. | Solyc11g069990.1 | 54578263 | 54580581 | | | |
| 42. | Solyc11g070000.1 | 54580666 | 54581469 | | | |
| 43. | Solyc11g070010.1 | 54583805 | 54585238 | | | |
| 44. | Solyc11g070020.1 | 54586315 | 54589900 | capana05g001982 | 191264072 | 191270402 |
| 45. | Solyc11g070030.1 | 54591888 | 54597480 | capana05g001981 | 191215249 | 191217529 |
| 46. | Solyc11g070040.1 | 54600090 | 54601526 | capana05g001980 | 191173688 | 191175500 |
| 47. | Solyc11g070050.1 | 54603351 | 54608716 | capana05g001977 | 191072407 | 191074667 |
| 48. | Solyc11g070060.1 | 54610400 | 54616034 | | | |
| 49. | Solyc11g070070.1 | 54617895 | 54619565 | capana05g001976 | 191056918 | 191058784 |
| 50. | Solyc11g070080.1 | 54621649 | 54625767 | capana05g001941 | 186116713 | 186156899 |
| 51. | Solyc11g070090.1 | 54627238 | 54632837 | | | |
| 52. | Solyc11g070100.1 | 54634568 | 54639038 | capana05g001932 | 184534345 | 184535319 |
| 53. | Solyc11g070110.1 | 54639947 | 54645583 | capana05g001927 | 183658207 | 183661606 |
| 54. | Solyc11g070120.1 | 54648188 | 54652538 | capana05g001926 | 183651665 | 183657915 |
| 55. | Solyc11g070130.1 | 54655309 | 54657842 | capana05g001925 | 183543506 | 183552490 |
| 56. | Solyc11g070140.1 | 54661689 | 54666936 | capana05g001918 | 183277146 | 183282105 |
| 57. | Solyc11g070150.1 | 54668129 | 54670734 | capana05g001897 | 180038455 | 180043799 |
| 58. | Solyc11g070160.1 | 54675157 | 54683874 | capana05g001891 | 179942588 | 179943580 |
| 59. | Solyc11g070170.1 | 54686417 | 54690867 | capana05g001886 | 179744890 | 179745201 |
| 60. | Solyc11g070180.1 | 54702280 | 54703011 | | | |
| 61. | Solyc11g070190.1 | 54704639 | 54705196 | | | |
| 62. | Solyc11g071190.1 | 54716880 | 54717890 | capana05g001882 | 179232190 | 179235642 |
| 63. | Solyc11g071200.1 | 54725737 | 54728330 | capana05g001880 | 179045755 | 179049136 |
| 64. | Solyc11g071210.1 | 54733490 | 54733669 | | | |
| 65. | Solyc11g071220.1 | 54744343 | 54748275 | | | |
| 66. | Solyc11g071230.1 | 54767748 | 54770501 | capana11g000020 | 248134 | 251415 |
| 67. | Solyc11g071240.1 | 54774080 | 54775706 | capana11g000079 | 2280596 | 2286141 |
| 68. | Solyc11g071250.1 | 54781753 | 54787477 | capana11g000018 | 227036 | 233096 |
| 69. | Solyc11g071260.1 | 54794646 | 54799169 | capana11g000077 | 2249772 | 2261134 |

**Table S3. Genes located within *Ty3* QTL region of tomato and their orthologs in pepper**

| S. No. | Genes in Pepper | Location in Tomato Chromosome 6 | | Ortholog in Pepper | Location of gene | |
|---|---|---|---|---|---|---|
| | | From | To | | From | To |
| 1. | Solyc06g051760.2 | 35365491 | 35374199 | Capana06g002469 | 167266983 | 167280593 |
| 2. | Solyc06g051770.1 | 35393201 | 35393755 | | | |
| 3. | Solyc06g051780.2 | 35440414 | 35456330 | | | |
| 4. | Solyc06g051790.2 | 35455312 | 35456522 | | | |
| 5. | Solyc06g051800.2 | 35478007 | 35479822 | | | |
| 6. | Solyc06g051810.2 | 35483484 | 35492863 | | | |
| 7. | Solyc06g051820.2 | 35499178 | 35505530 | | | |
| 8. | Solyc06g051830.1 | 35508188 | 35511766 | | | |
| 9. | Solyc06g051840.1 | 35553671 | 35554723 | Capana06g002704 | 193948417 | 193949565 |
| 10. | Solyc06g051850.1 | 35566326 | 35567963 | Capana06g002703 | 193855080 | 193859469 |
| 11. | Solyc06g051860.1 | 35569598 | 35571187 | | | |
| 12. | Solyc06g084760.1 | 35576515 | 35576694 | | | |
| 13. | Solyc06g084770.1 | 35576697 | 35576759 | | | |

**Table S4. Genes located within *Ty4* QTL region of tomato and their orthologs in pepper**

| S.No. | Gene_In Tomato | Location in Tomato | | Orthologs in pepper | Location in Pepper | |
|---|---|---|---|---|---|---|
| | | From | To | | From | To |
| 1. | Solyc03g019930.2 | 61283575 | 61286710 | capana03g001506 | 28289445 | 28290611 |
| 2. | Solyc03g019920.1 | 61287056 | 61287799 | capana03g001509 | 28301765 | 28302904 |
| 3. | Solyc03g019910.2 | 61287489 | 61291086 | capana03g001510 | 28328488 | 28329435 |
| 4. | Solyc03g019900.2 | 61293375 | 61300731 | capana03g001514 | 28408510 | 28410854 |
| 5. | Solyc03g019890.2 | 61302054 | 61311655 | capana03g001517 | 28446591 | 28447346 |
| 6. | Solyc03g019880.2 | 61315808 | 61318699 | capana03g001520 | 28484467 | 28488423 |
| 7. | Solyc03g019870.2 | 61323937 | 61327448 | capana03g001523 | 28518422 | 28525190 |
| 8. | Solyc03g019860.2 | 61328649 | 61329626 | | | |
| 9. | Solyc03g019850.2 | 61330650 | 61335362 | capana03g001524 | 28554242 | 28560359 |
| 10. | Solyc03g019840.2 | 61338765 | 61344140 | capana03g001525 | 28562466 | 28566194 |
| 11. | Solyc03g019830.2 | 61351114 | 61354088 | capana03g001526 | 28572926 | 28576191 |
| 12. | Solyc03g019820.2 | 61355168 | 61356506 | capana03g001527 | 28594724 | 28607416 |
| 13. | Solyc03g019810.2 | 61356600 | 61360277 | capana03g001528 | 28611737 | 28616589 |
| 14. | Solyc03g019800.2 | 61364240 | 61366038 | capana03g001536 | 28757947 | 28763792 |
| 15. | Solyc03g019790.2 | 61371475 | 61377262 | capana03g001537 | 28772030 | 28775379 |
| 16. | Solyc03g019780.2 | 61379181 | 61381390 | capana03g001538 | 28789606 | 28798393 |
| 17. | Solyc03g019770.2 | 61383048 | 61392365 | capana03g001539 | 28799836 | 28804106 |
| 18. | Solyc03g019760.2 | 61400646 | 61404690 | capana03g001540 | 28816102 | 28817304 |
| 19. | Solyc03g019750.2 | 61408666 | 61410195 | capana03g001546 | 28925594 | 28929033 |
| 20. | Solyc03g019740.1 | 61410380 | 61411075 | capana03g001547 | 28932272 | 28933204 |
| 21. | Solyc03g019730.2 | 61416754 | 61422688 | capana03g001549 | 29096393 | 29099761 |
| 22. | Solyc03g019720.2 | 61427543 | 61433968 | capana03g001552 | 29169758 | 29176580 |
| 23. | Solyc03g019710.2 | 61436784 | 61440333 | capana03g001553 | 29178394 | 29182680 |
| 24. | Solyc03g019700.1 | 61447870 | 61448079 | | | |
| 25. | Solyc03g019690.1 | 61452670 | 61453323 | capana03g001554 | 29342053 | 29344602 |
| 26. | Solyc03g019680.2 | 61457130 | 61463379 | capana06g000939 | 16528859 | 16560063 |
| 27. | Solyc03g019670.2 | 61465035 | 61468136 | | | |
| 28. | Solyc03g019660.2 | 61472882 | 61475414 | | | |
| 29. | Solyc03g019650.2 | 61480167 | 61484009 | | | |
| 30. | Solyc03g019640.1 | 61485874 | 61492187 | | | |
| 31. | Solyc03g019630.1 | 61495951 | 61496421 | | | |
| 32. | Solyc03g110840.1 | 61583623 | 61584111 | capana03g001462 | 27396244 | 27406782 |
| 33. | Solyc03g110850.1 | 61591009 | 61591500 | | | |
| 34. | Solyc03g110860.2 | 61594846 | 61599648 | capana03g001463 | 27409601 | 27421550 |
| 35. | Solyc03g110870.2 | 61600058 | 61600717 | | | |
| 36. | Solyc03g110880.2 | 61605476 | 61622076 | capana03g001461 | 27384039 | 27390475 |
| 37. | Solyc03g110890.1 | 61616069 | 61616974 | capana03g001460 | 27345034 | 27345543 |
| 38. | Solyc03g110900.2 | 61618311 | 61637433 | capana03g001459 | 27343306 | 27344645 |
| 39. | Solyc03g110910.2 | 61641949 | 61646431 | capana03g001458 | 27324590 | 27327966 |
| 40. | Solyc03g110920.2 | 61647388 | 61653891 | capana03g001457 | 27311350 | 27316153 |
| 41. | Solyc03g110930.2 | 61656827 | 61659918 | capana03g001456 | 27307479 | 27310315 |

| | | | | | | |
|---|---|---|---|---|---|---|
| 42. | Solyc03g110940.2 | 61662162 | 61670639 | capana03g001451 | 27233963 | 27247404 |
| 43. | Solyc03g110950.1 | 61674859 | 61676451 | capana03g001450 | 27227059 | 27228945 |
| 44. | Solyc03g110960.2 | 61677485 | 61680515 | capana03g001449 | 27215199 | 27219251 |
| 45. | Solyc03g110970.2 | 61680795 | 61682185 | capana03g001448 | 27212904 | 27214029 |
| 46. | Solyc03g110980.2 | 61684948 | 61687905 | capana03g001447 | 27208675 | 27211617 |
| 47. | Solyc03g110990.1 | 61690736 | 61690933 | | | |
| 48. | Solyc03g111000.2 | 61701908 | 61705478 | capana03g001445 | 27195701 | 27197908 |
| 49. | Solyc03g111010.2 | 61708516 | 61711998 | | | |
| 50. | Solyc03g111020.2 | 61712051 | 61713204 | | | |
| 51. | Solyc03g111030.2 | 61712633 | 61718529 | capana03g001444 | 27182301 | 27188338 |
| 52. | Solyc03g111040.1 | 61718834 | 61721182 | capana03g001442 | 27164589 | 27168655 |
| 53. | Solyc03g111050.2 | 61722801 | 61733152 | capana03g001397 | 25197043 | 25206522 |
| 54. | Solyc03g111060.2 | 61737916 | 61745047 | capana06g000949 | 16675242 | 16679817 |
| 55. | Solyc03g111070.2 | 61745070 | 61745823 | capana03g001400 | 25301763 | 25309466 |
| 56. | Solyc03g111080.2 | 61745766 | 61746741 | | | |
| 57. | Solyc03g111090.2 | 61746820 | 61753202 | capana03g001404 | 25529513 | 25535448 |
| 58. | Solyc03g111100.1 | 61766912 | 61767844 | capana03g001419 | 26873403 | 26874214 |
| 59. | Solyc03g111110.1 | 61788074 | 61788301 | | | |
| 60. | Solyc03g111120.2 | 61791871 | 61793259 | capana03g001420 | 26875708 | 26876352 |
| 61. | Solyc03g111130.1 | 61796294 | 61798483 | | | |
| 62. | Solyc03g111140.2 | 61799560 | 61802073 | | | |
| 63. | Solyc03g111150.2 | 61802707 | 61806299 | capana03g001422 | 26912533 | 26947516 |
| 64. | Solyc03g111160.2 | 61809555 | 61817635 | | | |
| 65. | Solyc03g111170.2 | 61817881 | 61823869 | capana03g001424 | 26951410 | 26952690 |

**Table S5. Genes located within *ty5* QTL region of tomato and their orthologs in pepper**

| S.No. | Name of Gene in Tomato | Location in Tomato | | Orthologs in Pepper | Location in Pepper | |
|---|---|---|---|---|---|---|
| | | From | To | | From | To |
| 1. | Solyc04g008510.2 | 2133538 | 2142268 | | | |
| 2. | Solyc04g008520.2 | 2142947 | 2150143 | capana05g000831 | 32034596 | 32038233 |
| 3. | Solyc04g008530.1 | 2154141 | 2155727 | capana05g000818 | 31841601 | 31843423 |
| 4. | Solyc04g008540.2 | 2157991 | 2165032 | capana05g000819 | 31846934 | 31860195 |
| 5. | Solyc04g008550.2 | 2166940 | 2170876 | capana05g000820 | 31862912 | 31869351 |
| 6. | Solyc04g008560.2 | 2173271 | 2179828 | capana05g000823 | 31875947 | 31882993 |
| 7. | Solyc04g008570.2 | 2182236 | 2184631 | capana03g001301 | 22834446 | 22837019 |
| 8. | Solyc04g008580.2 | 2185454 | 2191242 | capana05g000817 | 31834220 | 31839584 |
| 9. | Solyc04g008590.2 | 2195990 | 2201197 | capana05g000815 | 31787242 | 31789876 |
| 10. | Solyc04g008600.2 | 2202430 | 2209479 | capana05g000806 | 31583584 | 31583904 |
| 11. | Solyc04g008610.2 | 2212040 | 2228571 | capana03g001296 | 22695060 | 22697871 |
| 12. | Solyc04g008620.2 | 2231877 | 2239671 | capana05g000800 | 31405943 | 31407040 |
| 13. | Solyc04g008630.2 | 2242377 | 2248072 | capana05g000801 | 31444512 | 31446021 |
| 14. | Solyc04g008640.2 | 2253610 | 2275631 | | | |
| 15. | Solyc04g008650.2 | 2280193 | 2286296 | | | |

| | | | | | | |
|---|---|---|---|---|---|---|
| 16. | Solyc04g008660.2 | 2289220 | 2305938 | capana05g000802 | 31454847 | 31459759 |
| 17. | Solyc04g008670.1 | 2314687 | 2317056 | capana03g001342 | 24257343 | 24261796 |
| 18. | Solyc04g008680.2 | 2318621 | 2333205 | capana05g000805 | 31554382 | 31555314 |
| 19. | Solyc04g008690.2 | 2333617 | 2343002 | capana05g000798 | 31386835 | 31388625 |
| 20. | Solyc04g008700.2 | 2360494 | 2378751 | capana05g000794 | 31286240 | 31293951 |
| 21. | Solyc04g008710.2 | 2378787 | 2381066 | capana05g000793 | 31129828 | 31131616 |
| 22. | Solyc04g008720.2 | 2384699 | 2393112 | capana03g001292 | 22640037 | 22655941 |
| 23. | Solyc04g008730.2 | 2393747 | 2398009 | capana05g000781 | 30410870 | 30414288 |
| 24. | Solyc04g008740.2 | 2398964 | 2404186 | capana05g000778 | 30337103 | 30337669 |
| 25. | Solyc04g008750.2 | 2407781 | 2412048 | capana05g000768 | 30034470 | 30035227 |
| 26. | Solyc04g008760.1 | 2412878 | 2414011 | capana05g000767 | 30025138 | 30027468 |
| 27. | Solyc04g008770.2 | 2416241 | 2420323 | capana05g000766 | 29933832 | 29937546 |
| 28. | Solyc04g008780.2 | 2420696 | 2423766 | capana05g000764 | 29852729 | 29856127 |
| 29. | Solyc04g008790.1 | 2424815 | 2425648 | capana05g000760 | 29310086 | 29318151 |
| 30. | Solyc04g008800.2 | 2427020 | 2430714 | capana05g000757 | 29190520 | 29192984 |
| 31. | Solyc04g008810.2 | 2432009 | 2434241 | capana05g000755 | 29096508 | 29101902 |
| 32. | Solyc04g008820.2 | 2435267 | 2437869 | capana05g000754 | 29084738 | 29094635 |
| 33. | Solyc04g008830.1 | 2438053 | 2439834 | capana05g000753 | 29062757 | 29069830 |
| 34. | Solyc04g008840.2 | 2458319 | 2461577 | capana05g000752 | 28828605 | 28828907 |
| 35. | Solyc04g008850.1 | 2469802 | 2471241 | capana05g000748 | 28664381 | 28666288 |
| 36. | Solyc04g008860.2 | 2475731 | 2485874 | capana05g000747 | 28580194 | 28599518 |
| 37. | Solyc04g008870.2 | 2492193 | 2494187 | capana05g000746 | 28542902 | 28547536 |
| 38. | Solyc04g008880.2 | 2495076 | 2498241 | | | |
| 39. | Solyc04g008890.2 | 2499276 | 2502372 | | | |
| 40. | Solyc04g008900.2 | 2511229 | 2514610 | | | |
| 41. | Solyc04g008910.1 | 2519306 | 2528011 | | | |
| 42. | Solyc04g008920.1 | 2537116 | 2537840 | | | |
| 43. | Solyc04g008930.1 | 2547739 | 2548800 | | | |
| 44. | Solyc04g008940.2 | 2551125 | 2552678 | | | |
| 45. | Solyc04g008950.2 | 2554629 | 2556300 | | | |
| 46. | Solyc04g008960.2 | 2559465 | 2560652 | | | |
| 47. | Solyc04g008970.2 | 2564833 | 2566646 | | | |
| 48. | Solyc04g008980.2 | 2567073 | 2569411 | | | |
| 49. | Solyc04g008990.2 | 2573083 | 2578353 | | | |
| 50. | Solyc04g009000.1 | 2579236 | 2581365 | | | |
| 51. | Solyc04g009010.1 | 2582427 | 2583020 | | | |
| 52. | Solyc04g009020.2 | 2590565 | 2594701 | | | |
| 53. | Solyc04g009030.2 | 2597412 | 2600773 | | | |
| 54. | Solyc04g009040.2 | 2600952 | 2604337 | | | |
| 55. | Solyc04g009050.2 | 2611384 | 2617110 | | | |
| 56. | Solyc04g009060.2 | 2618806 | 2627468 | | | |
| 57. | Solyc04g009070.1 | 2629302 | 2629574 | | | |
| 58. | Solyc04g009080.1 | 2629628 | 2629996 | | | |
| 59. | Solyc04g009090.1 | 2630935 | 2632218 | | | |

| | | | | | | |
|---|---|---|---|---|---|---|
| 60. | Solyc04g009100.1 | 2632841 | 2633663 | | | |
| 61. | Solyc04g009110.1 | 2637079 | 2639610 | | | |
| 62. | Solyc04g009120.1 | 2641913 | 2644352 | | | |
| 63. | Solyc04g009130.2 | 2646823 | 2649379 | | | |
| 64. | Solyc04g009140.2 | 2651223 | 2653938 | | | |
| 65. | Solyc04g009150.1 | 2655518 | 2658052 | | | |
| 66. | Solyc04g009160.2 | 2660331 | 2668727 | | | |
| 67. | Solyc04g009170.1 | 2671422 | 2672351 | capana03g001962 | 39904102 | 39904887 |
| 68. | Solyc04g009180.1 | 2687517 | 2688317 | capana03g001963 | 39932053 | 39936190 |
| 69. | Solyc04g009190.2 | 2692465 | 2695221 | capana03g001964 | 39936201 | 39938909 |
| 70. | Solyc04g009200.2 | 2695320 | 2699163 | capana03g001966 | 39999757 | 40001103 |
| 71. | Solyc04g009210.1 | 2700811 | 2702490 | capana03g001967 | 40036805 | 40042289 |
| 72. | Solyc04g009220.1 | 2705636 | 2708194 | | | |
| 73. | Solyc04g009230.2 | 2708580 | 2711693 | capana03g001971 | 40470550 | 40472331 |
| 74. | Solyc04g009240.1 | 2713465 | 2716002 | | | |
| 75. | Solyc04g009250.1 | 2719885 | 2722407 | | | |
| 76. | Solyc04g009260.1 | 2728030 | 2730579 | | | |
| 77. | Solyc04g009270.2 | 2733652 | 2734643 | | | |
| 78. | Solyc04g009280.1 | 2734619 | 2734873 | | | |
| 79. | Solyc04g009290.1 | 2735950 | 2738484 | | | |
| 80. | Solyc04g009300.2 | 2738951 | 2744093 | | | |
| 81. | Solyc04g009310.2 | 2760682 | 2765405 | | | |
| 82. | Solyc04g009320.2 | 2766840 | 2771952 | | | |
| 83. | Solyc04g009330.2 | 2772793 | 2776023 | | | |
| 84. | Solyc04g009340.2 | 2776459 | 2785611 | | | |
| 85. | Solyc04g009350.2 | 2786259 | 2793145 | | | |
| 86. | Solyc04g009360.2 | 2793909 | 2799625 | | | |
| 87. | Solyc04g009370.2 | 2800746 | 2806524 | | | |
| 88. | Solyc04g009380.1 | 2807742 | 2815384 | | | |
| 89. | Solyc04g009390.1 | 2819264 | 2823743 | | | |
| 90. | Solyc04g009400.2 | 2827528 | 2833846 | | | |
| 91. | Solyc04g009410.2 | 2835286 | 2840148 | | | |
| 92. | Solyc04g009420.2 | 2840496 | 2842762 | | | |
| 93. | Solyc04g009430.2 | 2843670 | 2851326 | | | |
| 94. | Solyc04g009440.2 | 2856469 | 2858639 | | | |
| 95. | Solyc04g009450.1 | 2859789 | 2861588 | | | |
| 96. | Solyc04g009460.1 | 2864606 | 2864892 | | | |
| 97. | Solyc04g009470.2 | 2875483 | 2879317 | capana05g000745 | 28533618 | 28540821 |
| 98. | Solyc04g009480.1 | 2881453 | 2882162 | | | |
| 99. | Solyc04g009490.1 | 2909215 | 2910429 | capana05g000743 | 28463375 | 28463950 |
| 100. | Solyc04g009500.2 | 2915852 | 2927412 | | | |
| 101. | Solyc04g009510.2 | 2930038 | 2938296 | capana05g000741 | 28336919 | 28342819 |
| 102. | Solyc04g009520.2 | 2939506 | 2942143 | capana05g000740 | 28225239 | 28229380 |
| 103. | Solyc04g009530.2 | 2943884 | 2949090 | capana05g000733 | 27439799 | 27447868 |

| | | | | | | |
|---|---|---|---|---|---|---|
| 104. | Solyc04g009540.1 | 2950915 | 2951604 | capana05g000711 | 25853083 | 25865516 |
| 105. | Solyc04g009550.2 | 2955891 | 2960456 | capana05g000709 | 25804859 | 25807730 |
| 106. | Solyc04g009560.2 | 2961142 | 2970580 | capana05g000708 | 25742033 | 25767857 |
| 107. | Solyc04g009570.2 | 2972096 | 2974121 | | | |
| 108. | Solyc04g009580.2 | 2975791 | 2980078 | capana05g000706 | 25552082 | 25557475 |
| 109. | Solyc04g009590.2 | 2983229 | 2984644 | | | |
| 110. | Solyc04g009600.2 | 2991227 | 2993911 | capana05g000705 | 25510280 | 25517420 |
| 111. | Solyc04g009610.2 | 2996112 | 2997327 | capana05g000696 | 24069652 | 24071682 |
| 112. | Solyc04g009620.2 | 2998013 | 3006166 | capana05g000685 | 22990916 | 23003660 |
| 113. | Solyc04g009630.2 | 3009140 | 3012779 | capana05g000681 | 22752797 | 22762447 |
| 114. | Solyc04g009640.2 | 3012845 | 3016560 | capana05g000680 | 22748973 | 22751383 |
| 115. | Solyc04g009650.2 | 3019293 | 3024735 | | | |
| 116. | Solyc04g009660.2 | 3028988 | 3031950 | capana05g000653 | 21900621 | 21901763 |
| 117. | Solyc04g009670.1 | 3034824 | 3035351 | | | |
| 118. | Solyc04g009680.1 | 3035434 | 3036433 | | | |
| 119. | Solyc04g009690.1 | 3041461 | 3044007 | | | |
| 120. | Solyc04g009700.2 | 3045839 | 3049663 | capana05g000678 | 22718936 | 22725532 |
| 121. | Solyc04g009710.1 | 3049930 | 3051029 | capana05g000672 | 22661182 | 22666332 |
| 122. | Solyc04g009720.1 | 3052311 | 3055210 | capana05g000670 | 22630684 | 22633626 |
| 123. | Solyc04g009730.2 | 3056081 | 3060505 | capana12g002230 | 20752873<br>3 | 207535358 |
| 124. | Solyc04g009740.2 | 3062113 | 3072311 | capana05g000669 | 22572495 | 22577094 |
| 125. | Solyc04g009750.1 | 3074808 | 3075035 | | | |
| 126. | Solyc04g009760.1 | 3075183 | 3076217 | capana05g000668 | 22548291 | 22549418 |
| 127. | Solyc04g009770.2 | 3081668 | 3086277 | capana05g000666 | 22442067 | 22448067 |
| 128. | Solyc04g009780.1 | 3087597 | 3088157 | capana05g000665 | 22427318 | 22432442 |
| 129. | Solyc04g009790.2 | 3090794 | 3100196 | | | |
| 130. | Solyc04g009800.2 | 3108503 | 3114299 | capana05g000652 | 21881040 | 21892746 |
| 131. | Solyc04g009810.2 | 3117554 | 3125731 | capana05g000663 | 22406776 | 22411501 |
| 132. | Solyc04g009820.2 | 3139217 | 3143959 | capana05g000659 | 22070365 | 22073088 |
| 133. | Solyc04g009830.2 | 3154104 | 3161025 | capana05g000658 | 22060669 | 22063223 |
| 134. | Solyc04g009840.2 | 3163517 | 3167544 | capana05g000654 | 21949879 | 21951476 |
| 135. | Solyc04g009850.2 | 3167860 | 3170633 | | | |
| 136. | Solyc04g009860.2 | 3173261 | 3175305 | | | |
| 137. | Solyc04g009870.1 | 3184723 | 3184878 | | | |
| 138. | Solyc04g009880.1 | 3193245 | 3193652 | | | |
| 139. | Solyc04g009890.1 | 3195597 | 3197439 | | | |
| 140. | Solyc04g009900.2 | 3214698 | 3216087 | | | |
| 141. | Solyc04g009910.2 | 3230992 | 3232541 | | | |
| 142. | Solyc04g009920.2 | 3239955 | 3246719 | capana05g000644 | 21465586 | 21467626 |
| 143. | Solyc04g009930.1 | 3248530 | 3253636 | capana05g000548 | 14671952 | 14681114 |
| 144. | Solyc04g009940.2 | 3255363 | 3260014 | | | |
| 145. | Solyc04g009950.2 | 3261269 | 3266696 | capana05g000547 | 14661146 | 14665194 |
| 146. | Solyc04g009960.2 | 3280106 | 3284080 | capana05g000546 | 14637098 | 14644301 |
| 147. | Solyc04g009970.2 | 3283806 | 3292746 | capana05g000545 | 14616789 | 14620808 |

| | | | | | | |
|---|---|---|---|---|---|---|
| 148. | Solyc04g009980.2 | 3297717 | 3299388 | capana05g000544 | 14609977 | 14614464 |
| 149. | Solyc04g009990.2 | 3303089 | 3310711 | capana05g000543 | 14600077 | 14601694 |
| 150. | Solyc04g010000.2 | 3316295 | 3321417 | capana05g000542 | 14590987 | 14597648 |
| 151. | Solyc04g010010.1 | 3325753 | 3329213 | | | |
| 152. | Solyc04g010020.2 | 3333448 | 3338309 | | | |
| 153. | Solyc04g010030.1 | 3339704 | 3344083 | | | |
| 154. | Solyc04g010040.2 | 3347068 | 3350505 | | | |
| 155. | Solyc04g010050.2 | 3352129 | 3356470 | capana05g000541 | 14578525 | 14589272 |
| 156. | Solyc04g010060.2 | 3358228 | 3360403 | | | |
| 157. | Solyc04g010070.2 | 3365037 | 3378848 | capana05g000539 | 14519071 | 14523990 |
| 158. | Solyc04g010080.2 | 3379820 | 3388745 | capana05g000538 | 14488229 | 14490403 |
| 159. | Solyc04g010090.2 | 3391686 | 3396958 | capana05g000533 | 14348729 | 14349310 |
| 160. | Solyc04g010100.1 | 3397246 | 3397428 | | | |
| 161. | Solyc04g010110.2 | 3399938 | 3405925 | capana05g000531 | 14329920 | 14337842 |
| 162. | Solyc04g010120.2 | 3406497 | 3416361 | capana05g000518 | 13548972 | 13555573 |
| 163. | Solyc04g010130.1 | 3425360 | 3425635 | capana05g000517 | 13501556 | 13505098 |
| 164. | Solyc04g010140.1 | 3430046 | 3430213 | | | |
| 165. | Solyc04g010150.1 | 3431056 | 3431292 | | | |
| 166. | Solyc04g010160.1 | 3459669 | 3459902 | | | |
| 167. | Solyc04g010180.2 | 3502067 | 3502725 | capana05g000516 | 13439456 | 13444862 |
| 168. | Solyc04g010190.1 | 3508288 | 3508857 | capana05g000515 | 13429962 | 13430261 |
| 169. | Solyc04g010200.1 | 3520574 | 3522730 | capana05g000514 | 13415351 | 13428036 |
| 170. | Solyc04g010210.1 | 3525914 | 3528058 | | | |
| 171. | Solyc04g010220.2 | 3536536 | 3549046 | capana05g000513 | 13402564 | 13412576 |
| 172. | Solyc04g010230.2 | 3556402 | 3561755 | capana05g000508 | 13312128 | 13313162 |
| 173. | Solyc04g010240.2 | 3562006 | 3565042 | capana05g000506 | 13294924 | 13296029 |
| 174. | Solyc04g010250.2 | 3577656 | 3583500 | capana05g000503 | 13288383 | 13288871 |
| 175. | Solyc04g010260.2 | 3583577 | 3588628 | capana05g000502 | 13263338 | 13269140 |
| 176. | Solyc04g010270.1 | 3590492 | 3591691 | capana05g000501 | 13246752 | 13256089 |
| 177. | Solyc04g010280.1 | 3595816 | 3596083 | | | |
| 178. | Solyc04g010290.2 | 3618701 | 3622866 | capana05g000500 | 13201785 | 13208919 |
| 179. | Solyc04g010300.2 | 3626518 | 3628265 | capana05g000499 | 13160371 | 13167403 |
| 180. | Solyc04g010310.2 | 3631081 | 3639205 | | | |
| 181. | Solyc04g010320.1 | 3655025 | 3656521 | capana05g000498 | 13128364 | 13129630 |
| 182. | Solyc04g010330.2 | 3666239 | 3667699 | | | |
| 183. | Solyc04g011330.1 | 3722383 | 3722535 | | | |
| 184. | Solyc04g011340.2 | 3817607 | 3821614 | capana05g000461 | 10499154 | 10500458 |
| 185. | Solyc04g011350.2 | 3828961 | 3837844 | capana05g000457 | 10342255 | 10343729 |
| 186. | Solyc04g011360.2 | 3839267 | 3844110 | capana05g000409 | 9354533 | 9361462 |
| 187. | Solyc04g011370.2 | 3845935 | 3848508 | capana05g000408 | 9338570 | 9349829 |
| 188. | Solyc04g011380.2 | 3850974 | 3863049 | capana05g000407 | 9335666 | 9336775 |
| 189. | Solyc04g011390.1 | 3865521 | 3865832 | | | |
| 190. | Solyc04g011400.2 | 3868800 | 3872796 | capana05g000404 | 9277930 | 9279048 |
| 191. | Solyc04g011420.2 | 3881797 | 3883525 | capana05g000401 | 8870235 | 8875529 |

| | | | | | | |
|---|---|---|---|---|---|---|
| 192. | Solyc04g011430.2 | 3885139 | 3888726 | capana05g000399 | 8841922 | 8843148 |
| 193. | Solyc04g011440.2 | 3894918 | 3898067 | | | |
| 194. | Solyc04g011450.1 | 3899856 | 3900011 | | | |
| 195. | Solyc04g011460.1 | 3900842 | 3902368 | capana05g000398 | 8826233 | 8834235 |
| 196. | Solyc04g011470.1 | 3911982 | 3913070 | capana05g000396 | 8789962 | 8803795 |
| 197. | Solyc04g011480.2 | 3919695 | 3921556 | capana05g000397 | 8819321 | 8824261 |
| 198. | Solyc04g011490.2 | 3923552 | 3925091 | | | |
| 199. | Solyc04g011500.2 | 3937899 | 3939826 | capana05g000395 | 8785073 | 8789119 |
| 200. | Solyc04g011510.2 | 3944572 | 3949105 | capana05g000394 | 8772590 | 8777656 |
| 201. | Solyc04g011520.2 | 3956690 | 3961315 | capana05g000393 | 8762080 | 8765607 |
| 202. | Solyc04g011530.2 | 3961600 | 3968559 | capana05g000392 | 8744151 | 8750914 |
| 203. | Solyc04g011540.2 | 3971870 | 3974807 | | | |
| 204. | Solyc04g011550.2 | 3982374 | 3984026 | capana05g000391 | 8723379 | 8728796 |
| 205. | Solyc04g011560.2 | 3985755 | 3989214 | capana05g000390 | 8716240 | 8717262 |
| 206. | Solyc04g011570.2 | 3992355 | 3997255 | capana05g000388 | 8593830 | 8595017 |
| 207. | Solyc04g011580.2 | 3998019 | 4005142 | capana05g000387 | 8582411 | 8589940 |
| 208. | Solyc04g011590.2 | 4020984 | 4024553 | capana05g000386 | 8575166 | 8577259 |
| 209. | Solyc04g011600.2 | 4029691 | 4031884 | capana05g000385 | 8565714 | 8573155 |
| 210. | Solyc04g011610.1 | 4048148 | 4049335 | capana05g000384 | 8557537 | 8558262 |
| 211. | Solyc04g011620.2 | 4053218 | 4058441 | | | |
| 212. | Solyc04g011630.1 | 4103629 | 4104141 | capana05g000382 | 8283986 | 8288865 |
| 213. | Solyc04g011640.2 | 4104625 | 4105638 | | | |
| 214. | Solyc04g011650.2 | 4115805 | 4119204 | capana05g000383 | 8485090 | 8486935 |
| 215. | Solyc04g011660.1 | 4125296 | 4126710 | | | |
| 216. | Solyc04g011670.2 | 4150679 | 4155134 | capana05g000379 | 8196624 | 8197631 |
| 217. | Solyc04g011680.1 | 4155951 | 4157897 | | | |
| 218. | Solyc04g011690.2 | 4161778 | 4165327 | | | |
| 219. | Solyc04g011700.1 | 4166764 | 4167942 | capana05g000378 | 8179313 | 8183015 |
| 220. | Solyc04g011710.1 | 4176737 | 4177003 | | | |
| 221. | Solyc04g011720.2 | 4178949 | 4181437 | capana05g000374 | 8129168 | 8131271 |
| 222. | Solyc04g011730.2 | 4184455 | 4186832 | | | |
| 223. | Solyc04g011740.2 | 4195581 | 4197749 | capana05g000373 | 8121795 | 8127559 |
| 224. | Solyc04g011750.2 | 4202674 | 4203920 | | | |
| 225. | Solyc04g011760.2 | 4207599 | 4208104 | | | |
| 226. | Solyc04g011770.2 | 4219519 | 4221173 | | | |
| 227. | Solyc04g011780.1 | 4226837 | 4227145 | capana05g000372 | 8107817 | 8109458 |
| 228. | Solyc04g011790.1 | 4230288 | 4230596 | | | |
| 229. | Solyc04g011800.1 | 4233161 | 4233469 | | | |
| 230. | Solyc04g011810.1 | 4243506 | 4243814 | | | |
| 231. | Solyc04g011820.1 | 4247088 | 4247375 | | | |
| 232. | Solyc04g011830.1 | 4251562 | 4251870 | | | |
| 233. | Solyc04g011840.1 | 4255717 | 4256025 | | | |
| 234. | Solyc04g011850.1 | 4258018 | 4258326 | | | |
| 235. | Solyc04g011860.1 | 4274482 | 4274790 | | | |

| | | | | | | |
|---|---|---|---|---|---|---|
| 236. | Solyc04g011870.1 | 4277713 | 4278021 | | | |
| 237. | Solyc04g011880.1 | 4280042 | 4280350 | | | |
| 238. | Solyc04g011890.1 | 4283805 | 4284548 | | | |
| 239. | Solyc04g011900.2 | 4284893 | 4289460 | | | |
| 240. | Solyc04g011910.2 | 4291763 | 4293737 | | | |
| 241. | Solyc04g011920.1 | 4301985 | 4303535 | | | |
| 242. | Solyc04g011940.1 | 4311733 | 4313253 | | | |
| 243. | Solyc04g011950.1 | 4316238 | 4316501 | | | |
| 244. | Solyc04g011960.1 | 4319129 | 4321795 | | | |
| 245. | Solyc04g011970.1 | 4325923 | 4329809 | | | |
| 246. | Solyc04g011980.1 | 4339129 | 4341774 | | | |
| 247. | Solyc04g011990.1 | 4343284 | 4346090 | | | |
| 248. | Solyc04g012000.1 | 4347438 | 4347893 | | | |
| 249. | Solyc04g012010.2 | 4349137 | 4352103 | | | |
| 250. | Solyc04g012020.1 | 4352964 | 4354304 | | | |
| 251. | Solyc04g012030.2 | 4361229 | 4366187 | | | |
| 252. | Solyc04g012040.2 | 4367412 | 4375553 | | | |
| 253. | Solyc04g012050.2 | 4376146 | 4378819 | | | |
| 254. | Solyc04g012060.2 | 4384505 | 4390777 | | | |
| 255. | Solyc04g012070.1 | 4394391 | 4396124 | | | |
| 256. | Solyc04g012080.1 | 4399360 | 4400493 | | | |
| 257. | Solyc04g012090.1 | 4405884 | 4411075 | | | |
| 258. | Solyc04g012100.1 | 4412215 | 4415699 | | | |
| 259. | Solyc04g012110.1 | 4417380 | 4419708 | | | |
| 260. | Solyc04g012120.2 | 4428571 | 4430128 | | | |
| 261. | Solyc04g012130.1 | 4437198 | 4437386 | | | |
| 262. | Solyc04g012140.1 | 4438559 | 4438807 | | | |
| 263. | Solyc04g012150.2 | 4439296 | 4439701 | | | |
| 264. | Solyc04g012160.2 | 4446264 | 4449928 | | | |
| 265. | Solyc04g012170.2 | 4451581 | 4460764 | | | |
| 266. | Solyc04g012180.2 | 4461532 | 4467418 | | | |
| 267. | Solyc04g012190.1 | 4469507 | 4471607 | | | |
| 268. | Solyc04g012200.1 | 4477703 | 4477888 | | | |
| 269. | Solyc04g013200.1 | 4495150 | 4495647 | | | |
| 270. | Solyc04g014200.1 | 4507497 | 4508477 | | | |
| 271. | Solyc04g014210.2 | 4509385 | 4517601 | | | |
| 272. | Solyc04g014220.1 | 4521572 | 4522696 | | | |
| 273. | Solyc04g014230.1 | 4530800 | 4532108 | | | |
| 274. | Solyc04g014240.1 | 4542851 | 4544056 | | | |
| 275. | Solyc04g014250.2 | 4544956 | 4551102 | | | |
| 276. | Solyc04g014260.1 | 4560854 | 4561970 | | | |
| 277. | Solyc04g014270.2 | 4578308 | 4586276 | | | |
| 278. | Solyc04g014280.1 | 4587842 | 4588132 | capana05g000367 | 7972565 | 7982907 |
| 279. | Solyc04g014290.1 | 4590013 | 4590360 | | | |

| | | | | | | |
|---|---|---|---|---|---|---|
| 280. | Solyc04g014300.1 | 4596740 | 4596910 | | | |
| 281. | Solyc04g014310.1 | 4601261 | 4601464 | | | |
| 282. | Solyc04g014320.1 | 4605854 | 4606180 | | | |
| 283. | Solyc04g014330.1 | 4608760 | 4609086 | | | |
| 284. | Solyc04g014340.1 | 4610735 | 4610944 | | | |
| 285. | Solyc04g014350.1 | 4611641 | 4611817 | | | |
| 286. | Solyc04g014360.2 | 4614412 | 4616761 | capana05g000366 | 7968380 | 7971123 |
| 287. | Solyc04g014370.2 | 4624987 | 4631132 | capana05g000365 | 7950087 | 7952957 |
| 288. | Solyc04g014380.2 | 4632442 | 4636309 | capana05g000364 | 7929399 | 7931963 |
| 289. | Solyc04g014390.2 | 4638619 | 4641880 | | | |
| 290. | Solyc04g014400.2 | 4642331 | 4650160 | | | |
| 291. | Solyc04g014410.2 | 4654059 | 4656672 | | | |
| 292. | Solyc04g014420.2 | 4663745 | 4664930 | | | |
| 293. | Solyc04g014430.1 | 4665390 | 4670951 | | | |
| 294. | Solyc04g014440.2 | 4672029 | 4673081 | | | |
| 295. | Solyc04g014450.1 | 4673135 | 4679100 | | | |
| 296. | Solyc04g014460.2 | 4691348 | 4695742 | capana05g000363 | 7891105 | 7894343 |
| 297. | Solyc04g014470.2 | 4708995 | 4711600 | capana05g000361 | 7871870 | 7880664 |
| 298. | Solyc04g014480.2 | 4722700 | 4724263 | capana05g000358 | 7831476 | 7834568 |
| 299. | Solyc04g014490.1 | 4725303 | 4725698 | capana05g000356 | 7803106 | 7807295 |
| 300. | Solyc04g014500.2 | 4729086 | 4732903 | capana05g000355 | 7795312 | 7801401 |
| 301. | Solyc04g014510.2 | 4779960 | 4784217 | capana05g000354 | 7749815 | 7751326 |
| 302. | Solyc04g014520.1 | 4795021 | 4795914 | | | |
| 303. | Solyc04g014530.1 | 4802150 | 4802851 | capana05g000351 | 7666611 | 7669045 |
| 304. | Solyc04g014540.1 | 4814713 | 4815411 | capana05g000349 | 7618208 | 7620265 |
| 305. | Solyc04g014550.2 | 4821261 | 4824079 | | | |
| 306. | Solyc04g014560.2 | 4825799 | 4832573 | capana05g000347 | 7588260 | 7589956 |
