# Supplementary material for: Conservation and Dispersion of Genes Conferring Resistance to Tomato Begomoviruses between Tomato and Pepper Genomes
Source: Front Plant Sci. 2017 Nov 7;8:1803. doi: 10.3389/fpls.2017.01803 (PMC5681951; doi:10.3389/fpls.2017.01803)
Supplement: Supplementary file 2 [file Table2.pdf]

Table S6. Annotation of genes in *Ty1* qtl region of Tomato and their conserved orthologs in pepper

S. No.	Tomato Gene	Annotation in Tomato	Pepper Ortholog	Annotation in Pepper
1.	Solyc06g034130.2	LATE EMBRYOGENESIS ABUNDANT PROTEIN-LIKE PROTEIN	-	-
2.	Solyc06g034140.1	B3 DNA binding domain (B3 // Protein of unknown function (DUF724)	capana06g002534	SINA3_ARATH E3 ubiquitin-protein ligase
3.	Solyc06g034150.2	8-amino-7-oxononanoate synthase / AONS	capana06g002538	NUDC_MOUSE Nuclear migration protein
4.	Solyc06g034160.2	ubiquitin-like-conjugating enzyme ATG3 (ATG3	capana06g002539	KPYK_CLOAB Pyruvate kinase
5.	Solyc06g034170.1	ACYL-[ACYL-CARRIER-PROTEIN] DESATURASE 6, CHLOROPLASTIC		#N/A
6.	Solyc06g034180.1	AAA domain (AAA_17	capana06g002547	FOLC_BACSU Folylpolyglutamate synthase
7.	Solyc06g034190.1	Unknown Protein (AHRD V1)		#N/A
8.	Solyc06g034200.1	MFS transporter, PHS family, inorganic phosphate transporter (PHO84		#N/A
9.	Solyc06g034210.1	Unknown Protein (AHRD V1)		#N/A
10.	Solyc06g034220.2	VESICLE-ASSOCIATED PROTEIN 1-1-RELATED	capana06g002527	Y3475_ARATH Probable LRR receptor-like serine/threonine-protein kinase
11.	Solyc06g034230.1	F14O23.23 PROTEIN		#N/A
12.	Solyc06g034250.1	Unknown Protein (AHRD V1)		#N/A
13.	Solyc06g034260.1	F-box protein At2g35280 (AHRD V1 *-*-FB127_ARATH)		#N/A
14.	Solyc06g034290.2	GLYCEROL-3-PHOSPHATE TRANSPORTER 4-RELATED		#N/A
15.	Solyc06g034300.1	Unknown Protein (AHRD V1)		#N/A

16.	Solyc06g034310.2	DDE superfamily endonuclease (DDE_Tnp_4		#N/A
17.	Solyc06g034320.1	Domain of unknown function (DUF4535)		#N/A
18.	Solyc06g034330.2	autocrine motility factor receptor (AMFR, GP78)	capana06g002522	FAD3E_TOBAC Omega-3 fatty acid desaturase
19.	Solyc06g034340.1	No apical meristem (NAM protein)	capana06g002524	PLCD4_ARATH Phosphoinositide phospholipase
20.	Solyc06g034350.1	Unknown Protein (AHRD V1)		#N/A
21.	Solyc06g034360.1	PECTINESTERASE/PECTINESTERASE INHIBITOR 33-RELATED	capana06g002526	FLS2_ARATH LRR receptor-like serine/threonine-protein kinase
22.	Solyc06g034370.1	INVERTASE/PECTIN METHYLESTERASE INHIBITOR FAMILY PROTEIN / DC 1.2-LIKE PROTEIN-RELATED		#N/A
23.	Solyc06g034380.1	60S/50S RIBOSOMAL PROTEIN L6/L9 // SUBFAMILY NOT NAMED		#N/A
24.	Solyc06g034390.1	Phosphate-induced protein 1 conserved region (Phi_1		#N/A
25.	Solyc06g034400.1	Unknown Protein (AHRD V1)		#N/A
26.	Solyc06g034410.2	Protein of unknown function (DUF1442)		#N/A
27.	Solyc06g035410.1	PURINE PERMEASE 1-RELATED		#N/A
28.	Solyc06g035420.1	PURINE PERMEASE 1-RELATED		#N/A
29.	Solyc06g035430.2	AT HOOK MOTIF DNA-BINDING FAMILY PROTEIN-RELATED		#N/A
30.	Solyc06g035440.2	TARGETING PROTEIN FOR XKLP2 // SUBFAMILY NOT NAMED		#N/A
31.	Solyc06g035450.2	DEAD-BOX ATP-DEPENDENT RNA HELICASE 7		#N/A
32.	Solyc06g035460.2	DEAD-BOX ATP-DEPENDENT RNA HELICASE 7		#N/A

33.	Solyc06g035470.1	MuDR family transposase		#N/A
34.	Solyc06g035480.2	EPIDIDYMAL MEMBRANE PROTEIN E9-RELATED // SUBFAMILY NOT NAMED		#N/A
35.	Solyc06g035490.2	TRANSCRIPTION FACTOR BHLH118-RELATED		#N/A
36.	Solyc06g035500.1	Unknown Protein (AHRD V1)		#N/A
37.	Solyc06g035510.1	Unknown Protein (AHRD V1)		#N/A
38.	Solyc06g035520.2	CARBOXYLESTERASE 120-RELATED		#N/A
39.	Solyc06g035530.2	gibberellin 20-oxidase (E1.14.11.12		#N/A
40.	Solyc06g035540.1	Unknown Protein (AHRD V1)		#N/A
41.	Solyc06g035550.2	Unknown Protein (AHRD V1)		#N/A
42.	Solyc06g035560.1	Unknown Protein (AHRD V1)		#N/A
43.	Solyc06g035570.1	MADS BOX PROTEIN		#N/A
44.	Solyc06g035580.2	GLUCOSE-METHANOL-CHOLINE (GMC	capana01g000403	#N/A
45.	Solyc06g035590.1	Unknown Protein (AHRD V1)		#N/A
46.	Solyc06g035600.2	PPR repeat (PPR // Ribosomal Proteins L2, C-terminal domain	capana01g000397	PHR_ARATH Deoxyribodipyrimidine photo-lyase
47.	Solyc06g035610.2	RNA recognition motif. (a.k.a. RRM, RBD, or RNP domain (RRM_1)		#N/A
48.	Solyc06g035620.2	GRAS domain family (GRAS		#N/A
49.	Solyc06g035630.1	Unknown Protein (AHRD V1)		#N/A
50.	Solyc06g035640.1	GAG-POL-RELATED RETROTRANSPOSON // SUBFAMILY NOT NAMED		#N/A
51.	Solyc06g035650.1	Unknown Protein (AHRD V1)		#N/A
52.	Solyc06g035660.1	Ulp1 protease family, C-terminal catalytic domain (Peptidase_C48		#N/A
53.	Solyc06g035670.1	Unknown Protein (AHRD V1)		#N/A

54.	Solyc06g035680.1	Sorbitol-6-phosphate 2-dehydrogenase / Ketosephosphate reductase		#N/A
55.	Solyc06g035690.2	PER1-RELATED	capana03g002393	PGAP3_XENTR Post-GPI attachment to proteins factor 3
56.	Solyc06g035700.1	ETHYLENE-RESPONSIVE TRANSCRIPTION FACTOR ERF025-RELATED	capana03g002388	DRE1C_ARATH Dehydration-responsive element-binding protein
57.	Solyc06g035710.1	MATE EFFLUX FAMILY PROTEIN	capana01g000327	YD338_YEAST Uncharacterized transporter YDR338C
58.	Solyc06g035720.2	BAG FAMILY MOLECULAR CHAPERONE REGULATOR 2-RELATED	capana01g000326	BAG1_ARATH BAG family molecular chaperone regulator
59.	Solyc06g035740.1	SWIM zinc finger (SWIM // MULE transposase domain (MULE)	capana03g002469	FUCO1_ARATH Alpha-L-fucosidase
60.	Solyc06g035750.1	Unknown Protein (AHRD V1)		#N/A
61.	Solyc06g035760.2	CYTOCHROME P450 MONOOXYGENASE-LIKE PROTEIN	capana01g000367	C93A2_SOYBN Cytochrome P450 93A2
62.	Solyc06g035770.1	Unknown Protein (AHRD V1)		#N/A
63.	Solyc06g035790.2	METHYLTRANSFERASE // SUBFAMILY NOT NAMED		#N/A
64.	Solyc06g035800.1	Unknown Protein (AHRD V1)		#N/A
65.	Solyc06g035810.1	Putative S-adenosyl-L-methionine-dependent methyltransferase (Methyltransf_29		#N/A
66.	Solyc06g035820.1	PROTEASE INHIBITOR/SEED STORAGE/LIPID TRANSFER PROTEIN (LTP FAMILY PROTEIN)		#N/A
67.	Solyc06g035830.1	Unknown Protein (AHRD V1)		#N/A
68.	Solyc06g035840.1	Unknown Protein (AHRD V1)		#N/A
69.	Solyc06g035850.1	Unknown Protein (AHRD V1)		#N/A

70.	Solyc06g035860.1	Unknown Protein (AHRD V1)		#N/A
71.	Solyc06g035870.2	MEMBRANE STEROID-BINDING PROTEIN 1-RELATED		#N/A
72.	Solyc06g035880.1	HOMEOBOX-LEUCINE ZIPPER PROTEIN HDG7		#N/A
73.	Solyc06g035890.1	Unknown Protein (AHRD V1)		#N/A
74.	Solyc06g035900.1	REMORIN FAMILY PROTEIN-RELATED		#N/A
75.	Solyc06g035910.1	Remorin, C-terminal region (Remorin_C		#N/A
76.	Solyc06g035920.2	Remorin, N-terminal region (Remorin_N		#N/A
77.	Solyc06g035930.1	Unknown Protein (AHRD V1)		#N/A
78.	Solyc06g035940.2	homeobox-leucine zipper protein (HD-ZIP		#N/A
79.	Solyc06g035960.2	Oxalate--CoA ligase / Oxalyl-CoA synthetase		#N/A
80.	Solyc06g035970.2	TUBULIN // SUBFAMILY NOT NAMED		#N/A
81.	Solyc06g035980.2	ACTIN-DEPOLYMERIZING FACTOR 10-RELATED		#N/A
82.	Solyc06g035990.2	RNA POLYMERASE III DNA DIRECTED -RELATED		#N/A
83.	Solyc06g036000.2	DNA-directed RNA polymerase III subunit RPC3 (RPC3, POLR3C		#N/A
84.	Solyc06g036010.1	0		#N/A
85.	Solyc06g036020.1	0		#N/A
86.	Solyc06g036030.1	0		#N/A
87.	Solyc06g036040.1	0		#N/A
88.	Solyc06g036050.2	Large subunit ribosomal protein L36e (RP-L36e, RPL36		#N/A
89.	Solyc06g036060.2	C2H2-LIKE ZINC FINGER PROTEIN		#N/A
90.	Solyc06g036070.2	SKP1-LIKE PROTEIN 20-RELATED		#N/A
91.	Solyc06g036080.2	MITOGEN-ACTIVATED KINASE KINASE KINASE // SUBFAMILY NOT		#N/A

		NAMED		
92.	Solyc06g036090.1	Unknown Protein (AHRD V1)		#N/A
93.	Solyc06g036100.2	Sodium sulfate symporter and related arsenite permeases		#N/A
94.	Solyc06g036110.1	C2 domain		#N/A
95.	Solyc06g036120.2	Unknown Protein (AHRD V1)		#N/A
96.	Solyc06g036130.2	MATE EFFLUX FAMILY PROTEIN	capana03g002183	TT12_ARATH Protein TRANSPARENT TESTA 12
97.	Solyc06g036140.1	RNA-directed DNA polymerase / Revertase		#N/A
98.	Solyc06g036150.1	SENTRIN/SUMO-SPECIFIC PROTEASE // SUBFAMILY NOT NAMED		#N/A
99.	Solyc06g036170.1	SCARECROW-LIKE PROTEIN 8	capana03g002179	SCL8_ARATH Scarecrow-like protein
100.	Solyc06g036180.2	Unknown Protein (AHRD V1)		#N/A
101.	Solyc06g036190.1	MITOGEN-ACTIVATED PROTEIN KINASE KINASE KINASE 19-RELATED		#N/A
102.	Solyc06g036200.1	TRANSCRIPTION INITIATION FACTOR TFIID/SUPT3-RELATED		#N/A
103.	Solyc06g036210.1	NAD(PH-QUINONE OXIDOREDUCTASE SUBUNIT K, CHLOROPLASTIC		#N/A
104.	Solyc06g036220.1	NADPH OXIDASE // SUBFAMILY NOT NAMED	capana03g002234	KIF22_PONAB Kinesin-like protein
105.	Solyc06g036230.2	Unknown Protein (AHRD V1)		#N/A
106.	Solyc06g036240.1	ATP-BINDING CASSETTE TRANSPORTER // SUBFAMILY NOT NAMED		#N/A
107.	Solyc06g036250.1	Unknown Protein (AHRD V1)		#N/A

108.	Solyc06g036260.2	Beta-carotene 3-hydroxylase / Beta-carotene 3,3'-monooxygenase		
109.	Solyc06g036270.1	Ulp1 protease family, C-terminal catalytic domain (Peptidase_C48		
110.	Solyc06g036290.2	HEAT SHOCK PROTEIN 90 // SUBFAMILY NOT NAMED		
111.	Solyc06g036300.2	SWI/SNF COMPLEX-RELATED // SUBFAMILY NOT NAMED		#N/A
112.	Solyc06g036310.2	COPPER TRANSPORT FAMILY PROTEIN-RELATED		
113.	Solyc06g036320.1	Protein-serine/threonine phosphatase / Serine/threonine specific protein phosphatase		#N/A
114.	Solyc06g036330.1	Thiomorpholine-carboxylate dehydrogenase / Ketimine reductase		#N/A
115.	Solyc06g036340.2	PENTATRICOPEPTIDE REPEAT-CONTAINING PROTEIN-LIKE PROTEIN		
116.	Solyc06g036350.2	NADH dehydrogenase (ubiquinone 1 alpha subcomplex subunit 5 (NDUFA5)		#N/A
117.	Solyc06g036360.1	Unknown Protein (AHRD V1)		#N/A
118.	Solyc06g036370.1	Unknown Protein (AHRD V1)		#N/A
119.	Solyc06g036380.1	Unknown Protein (AHRD V1)		#N/A
120.	Solyc06g036390.1	Unknown Protein (AHRD V1)		#N/A
121.	Solyc06g036410.1	PROTEIN SGT1 HOMOLOG A-RELATED		#N/A
122.	Solyc06g036420.1	Suppressor of G2 allele of SKP1 (SUGT1, SGT1)		#N/A
123.	Solyc06g036430.2	RETICULON-LIKE PROTEIN B5-RELATED		#N/A
124.	Solyc06g036440.1	S-TYPE ANION CHANNEL SLAH2-RELATED		#N/A

125.	Solyc06g036450.1	ETHYLENE RESPONSE SENSOR 2-RELATED		#N/A
126.	Solyc06g036460.2	MEMBRANE-ASSOCIATED KINASE REGULATOR 6-RELATED		#N/A
127.	Solyc06g036470.1	Protein kinase domain (Pkinase // S-locus glycoprotein domain (S_locus_glycoprotein)		#N/A
128.	Solyc06g036480.1	ABC TRANSPORTER C FAMILY MEMBER 10		#N/A
129.	Solyc06g036490.1	ATP-binding cassette, subfamily C (CFTR/MRP, member 2)		#N/A
130.	Solyc06g036500.1	Unknown Protein (AHRD V1)		#N/A
131.	Solyc06g036510.1	Ulp1 protease family, C-terminal catalytic domain (Peptidase_C48		#N/A
132.	Solyc06g036520.1	Unknown Protein (AHRD V1)		#N/A
133.	Solyc06g036530.1	Protein of unknown function (DUF 659)		#N/A
134.	Solyc06g036540.1	Unknown Protein (AHRD V1)		#N/A
135.	Solyc06g036550.1	Unknown Protein (AHRD V1)		#N/A
136.	Solyc06g036570.1	Unknown Protein (AHRD V1)		#N/A
137.	Solyc06g036580.2	U4/U6 small nuclear ribonucleoprotein PRP3 (PRPF3, PRP3		#N/A
138.	Solyc06g036590.1	Unknown Protein (AHRD V1)		#N/A
139.	Solyc06g036600.1	Alternative splicing factor SRp55/B52/SRp75 (RRM superfamily// Spliceosomal protein snRNP-U1A/U2B		#N/A
140.	Solyc06g036610.1	KRAB-A DOMAIN-CONTAINING PROTEIN 2		#N/A
141.	Solyc06g036620.1	ANKYRIN REPEAT-CONTAINING PROTEIN		#N/A
142.	Solyc06g036630.1	ANKYRIN REPEAT-CONTAINING PROTEIN		#N/A
143.	Solyc06g036640.2	ALPHA- 1,6 -		#N/A

		FUCOSYLTRANSFERASE		
144.	Solyc06g036650.1	Unknown Protein (AHRD V1)		#N/A
145.	Solyc06g036660.1	Unknown Protein (AHRD V1)		#N/A
146.	Solyc06g036670.1	UNCHARACTERIZED PUTATIVE METHYLTRANSFERASE // SUBFAMILY NOT NAMED		#N/A
147.	Solyc06g036680.1	Unknown Protein (AHRD V1)		#N/A
148.	Solyc06g036690.1	Unknown Protein (AHRD V1)		#N/A
149.	Solyc06g036700.1	Histone-lysine N-methyltransferase SETD3 [EC:2.1.1.43] (SETD3		#N/A
150.	Solyc06g036710.1	Unknown Protein (AHRD V1)		#N/A
151.	Solyc06g036720.2	SCYTHE/BAT3		#N/A
152.	Solyc06g036750.1	REVERSE TRANSCRIPTASE/RNA-DEPENDENT DNA POLYMERASE-RELATED		#N/A
153.	Solyc06g036770.1	Unknown Protein (AHRD V1)		#N/A
154.	Solyc06g036780.2	(AHRD V1 *-*- Q0JJY9_ORYSJ); contains Interpro domain(s) IPR000620 Protein of unknown function DUF6, transmembrane		#N/A
155.	Solyc06g036790.1	Unknown Protein (AHRD V1)		#N/A
156.	Solyc06g036800.1	Unknown Protein (AHRD V1)		#N/A
157.	Solyc06g036810.1	Domain of unknown function (DUF4216)		#N/A
158.	Solyc06g036820.1	Viral movement protein (MP)		#N/A
159.	Solyc06g036850.1	Unknown Protein (AHRD V1)		#N/A
160.	Solyc06g036890.1	Endonuclease/exonuclease/phosphatase (AHRD V1 *-*- A2Q500_MEDTR)		#N/A
161.	Solyc06g043370.1	Plant transposase (Ptta/En/Spm family (Transposase_24)		#N/A
162.	Solyc06g043360.1	3-methyl-2-oxobutanoate dehydrogenase (2-methylpropanoyl-transferring/ Dehydrogenase, branched chain alpha-		#N/A

		keto acid		
163.	Solyc06g043350.1	Unknown Protein (AHRD V1)		#N/A
164.	Solyc06g043340.1	Unknown Protein (AHRD V1)		#N/A
165.	Solyc06g043320.1	Unknown Protein (AHRD V1)		#N/A
166.	Solyc06g043280.1	Unknown Protein (AHRD V1)		#N/A
167.	Solyc06g043270.1	SKP1 // SUBFAMILY NOT NAMED		#N/A
168.	Solyc06g043260.1	Unknown Protein (AHRD V1)		#N/A
169.	Solyc06g043250.2	Protein kinase domain (Pkinase // Universal stress protein family (Usp)		#N/A
170.	Solyc06g043240.1	Unknown Protein (AHRD V1)		#N/A
171.	Solyc06g043230.1	SWIM zinc finger		#N/A
172.	Solyc06g043210.1	HECT Ubiquitin (AHRD V1 *-*- Q2HT48_MEDTR)		#N/A
173.	Solyc06g043180.1	Ulp1 protease family C-terminal catalytic domain containing protein		#N/A
174.	Solyc06g043170.2	Actin-related protein 5 (ACTR5, ARP5, INO80M)		#N/A
175.	Solyc06g043160.1	PRE-MRNA SPLICING FACTOR PRP8		#N/A

Table S7. Annotation of genes in *Ty2* qtl region of Tomato and their conserved orthologs in pepper

S.No	Tomato gene	Annotation in Tomato	Pepper ortholog	Annotation in Pepper
1.	Solyc11g069590	Receptor-like protein kinase	capana11g000063	PERK2_ARATH Proline-rich receptor-like protein kinase
2.	Solyc11g069600	Inter-alpha-trypsin inhibitor heavy chain H4	capana11g000100	MGP_ARATH Zinc finger protein MAGPIE
3.	Solyc11g069610	Chromatin remodeling complex subunit	capana11g000099	FLA11_ARATH Fasciclin-like

		(AHRD V1 *--- B9N465_POPTR)		arabinogalactan protein
4.	Solyc11g069620	Cc-nbs-lrr%2C resistance protein	capana05g001827	R13L1_ARATH Putative disease resistance RPP13-like protein
5.	Solyc11g069630	Receptor-like protein kinase		#N/A
6.	Solyc11g069640	Carbonic anhydrase family protein (	capana11g000062	NEC3_NICLS Bifunctional monodehydroascorbate reductase and carbonic anhydrase nectarin-3
7.	Solyc11g069650	Unknown Protein (AHRD V1)		#N/A
8.	Solyc11g069660	Nbs-lrr%2C resistance protein		#N/A
9.	Solyc11g069670	Disease resistance protein R3a-like protein (Fragment) (AHRD V1 ***-B3F583_SOLDE)		#N/A
10.	Solyc11g069680	Acyltransferase-like protein (		#N/A
11.	Solyc11g069690	Protein disulfide isomerase		#N/A
12.	Solyc11g069700	Elongation factor 1-alpha (IPR004539 Translation elongation factor	capana11g000089	YRDC_RAT YrdC domain-containing protein
13.	Solyc11g069710	ABC transporter G family member 3	capana05g002016	PM34_DICDI Mitochondrial substrate carrier family protein
14.	Solyc11g069720	26S protease regulatory subunit 6B homolog	capana05g002005	DCR_ARATH BAHD acyltransferase
15.	Solyc11g069730	Unknown Protein (AHRD V1)		#N/A
16.	Solyc11g069740	Nitrate transporter	capana11g000042	NRT24_ARATH High affinity nitrate transporter
17.	Solyc11g069750	Nitrate transporter		#N/A
18.	Solyc11g069760	High affinity nitrate transporter protein		#N/A
19.	Solyc11g069770	Transcription factor MADS		#N/A
20.	Solyc11g069780	2-phosphoglycerate kinase		#N/A
21.	Solyc11g069790	chaperonin	capana11g000039	RUBA_PEA RuBisCO large subunit-binding protein subunit alpha
22.	Solyc11g069800	cytochrome P450	capana11g000083	#N/A
23.	Solyc11g069810	OTU domain containing protein	capana11g000082	PIP21_ORYSJ Probable aquaporin
24.	Solyc11g069820	ABC transporter G family member 28	capana05g002017	#N/A

25.	Solyc11g069830	Arsenite ATPase transporter (Eurofung)		#N/A
26.	Solyc11g069840	Os03g0859900 protein	capana11g000091	C3H24_ARATH Zinc finger CCCH domain-containing protein
27.	Solyc11g069850	Telomere repeat-binding protein 4	capana11g000092	#N/A
28.	Solyc11g069860	Glutaredoxin	capana11g000093	FK132_ARATH F-box/kelch-repeat protein
29.	Solyc11g069870	Ripening-related protein 3	capana11g000094	Y0010_DICDI Probable serine/threonine-protein kinase
30.	Solyc11g069880	Ripening-related protein 3		#N/A
31.	Solyc11g069890	BEL1-like homeodomain protein 8	capana11g000095	PDX2_ARATH Pyridoxal biosynthesis protein
32.	Solyc11g069900	Unknown Protein (AHRD V1)		#N/A
33.	Solyc11g069910	DNA-directed RNA polymerase II subunit J	capana11g000096	#N/A
34.	Solyc11g069920	Nbs%2C resistance protein fragment	capana05g002018	COI1_ARATH Coronatine-insensitive protein 1
35.	Solyc11g069930	Disease resistance protein R3a-like protein		#N/A
36.	Solyc11g069940	Glutaredoxin	capana05g002010	DCR_ARATH BAHD acyltransferase SV=1
37.	Solyc11g069950	Cell division protease ftsH homolog		#N/A
38.	Solyc11g069960	Receptor like kinase%2C RLK	capana05g001985	PT311_ARATH Probable sugar phosphate/phosphate translocator
39.	Solyc11g069970	Harpin-induced 1	capana05g001984	SF3_HELAN Pollen-specific protein
40.	Solyc11g069980	Interferon-induced GTP-binding protein Mx (Fragment)	capana05g001983	Y1500_ARATH Probable tyrosine-protein phosphatase
41.	Solyc11g069990	Nbs-lrr%2C resistance protein		#N/A
42.	Solyc11g070000	Nbs%2C resistance protein fragment		#N/A
43.	Solyc11g070010	F8A5.6 protein (AHRD V1 **-- Q9ZP57_ARATH)		#N/A
44.	Solyc11g070020	Unknown Protein (AHRD V1)	capana05g001982	PLCD2_ARATH Phosphoinositide phospholipase
45.	Solyc11g070030	NADH-quinone oxidoreductase subunit B	capana05g001981	#N/A
46.	Solyc11g070040	Pentatricopeptide repeat-containing protein	capana05g001980	G3OX1_ARATH Gibberellin 3-beta-

				dioxygenase 1
47.	Solyc11g070050	DUF866 domain protein	capana05g001977	PPIL4_NEUCR Peptidyl-prolyl cis-trans isomerase-like 4
48.	Solyc11g070060	DUF866 domain protein		#N/A
49.	Solyc11g070070	Zinc finger CCCH domain-containing protein	capana05g001976	#N/A
50.	Solyc11g070080	Carboxyl-terminal peptidase	capana05g001941	SMC6_HUMAN Structural maintenance of chromosomes protein
51.	Solyc11g070090	Carboxyl-terminal peptidase		#N/A
52.	Solyc11g070100	Early flowering 3)	capana05g001932	CXE13_ARATH Probable carboxylesterase
53.	Solyc11g070110	Epsin	capana05g001927	#N/A
54.	Solyc11g070120	Charged multivesicular body protein	capana05g001926	TDR_ARATH Leucine-rich repeat receptor-like protein kinase
55.	Solyc11g070130	Profilin	capana05g001925	RFT1_XENTR Protein RFT1 homolog
56.	Solyc11g070140	Cell division protein kinase 2	capana05g001918	PUB35_ARATH U-box domain-containing protein
57.	Solyc11g070150	Histidine phosphotransfer protein	capana05g001897	CNR6_MAIZE Cell number regulator 6
58.	Solyc11g070160	Os06g0483900 protein (Fragment)	capana05g001891	CXE1_ACTER Carboxylesterase 1
59.	Solyc11g070170	Casein kinase I	capana05g001886	GRXS6_ARATH Monothiol glutaredoxin-S6
60.	Solyc11g070180	Unknown Protein (AHRD V1)		#N/A
61.	Solyc11g070190	Unknown Protein (AHRD V1)		#N/A
62.	Solyc11g071190	C3HC4 type zinc-finger domain-containing protein	capana05g001882	Y4885_ARATH Probable LRR receptor-like serine/threonine-protein kinase
63.	Solyc11g071200	Unknown Protein (AHRD V1)	capana05g001880	#N/A
64.	Solyc11g071210	Unknown Protein (AHRD V1)%3B contains Interpro domain(s) IPR005455 Profilin%2C plant		#N/A
65.	Solyc11g071220	Os05g0596200 protein (Fragment) (AHRD V1 *-*- Q0DFE0_ORYSJ)		#N/A

66.	Solyc11g071230	Galactosylgalactosylxylosylprotein 3-beta-glucuronosyltransferase 1	capana11g000020	IRX9H_ARATH Probable beta-1,4-xylosyltransferase
67.	Solyc11g071240	U-box domain-containing protein	capana11g000079	REV_ARATH Homeobox-leucine zipper protein
68.	Solyc11g071250	EMBYO FLOWERING 1-like protein	capana11g000018	EMF1_ARATH Protein EMBRYONIC FLOWER 1
69.	Solyc11g071260	Ubiquitin-conjugating enzyme E2	capana11g000077	PP6R3_HUMAN Serine/threonine-protein phosphatase 6 regulatory subunit 3

Table S8. Annotation of genes in *Ty3* qtl region of Tomato and their conserved orthologs in pepper

S.No	Tomato gene	Annotation in Tomato	Pepper ortholog	Annotation in Pepper
1.	Solyc06g051760	Arginyl-tRNA--protein transferase 1	Capana06g002469	MYOB_DICDI Myosin IB heavy chain
2.	Solyc06g051770	Unknown Protein (AHRD V1)		
3.	Solyc06g051780	Poly(A) RNA polymerase		
4.	Solyc06g051790	Unknown Protein (AHRD V1)		
5.	Solyc06g051800	Expansin		
6.	Solyc06g051810	X1 (Fragment) Region of unknown function XS		
7.	Solyc06g051820	Myosin		
8.	Solyc06g051830	Potassium transporter 17		
9.	Solyc06g051840	Ethylene responsive transcription factor 1a	Capana06g002704	RING1_GOSHI E3 ubiquitin-protein ligase
10.	Solyc06g051850	Inorganic phosphate transporter 6	Capana06g002703	-
11.	Solyc06g05186	Inorganic phosphate transporter 6		

	0			
12.	Solyc06g08476 0	Cytochrome P450 monooxygenase CYP72A68		
13.	Solyc06g08477 0	Unknown Protein (AHRD V1)		

Table S9. Annotation of genes in *Ty4* qtl region of Tomato and their conserved orthologs in pepper

S.No	Tomato gene	Annotation in Tomato	Pepper ortholog	Annotation in Pepper
1.	Solyc03g019930	UPF0415 protein C7orf25 homolog	capana03g001506	RNG1L_ARATH E3 ubiquitin-protein ligase
2.	Solyc03g019920	Harpin-induced protein-like (Fragment)	capana03g001509	FB341_ARATH F-box protein
3.	Solyc03g019910	Unknown Protein (AHRD V1)	capana03g001510	-
4.	Solyc03g019900	Serine/threonine-protein phosphatase	capana03g001514	Probable ADP-ribosylation factor GTPase-activating protein
5.	Solyc03g019890	Beta-galactosidase (	capana03g001517	
6.	Solyc03g019880	UPF0426 protein	capana03g001520	
7.	Solyc03g019870	Cytochrome P450	capana03g001523	TBCB_CAEEL Tubulin-specific chaperone B
8.	Solyc03g019860	Unknown Protein (AHRD V1)		
9.	Solyc03g019850	MAP kinase	capana03g001524	YR106_MIMIV Uncharacterized protein
10	Solyc03g019840	Plastid DNA-binding protein (	capana03g001525	NAC74_ORYSJ NAC domain-containing protein
11	Solyc03g019830	Receptor like kinase%2C RLK	capana03g001526	HDG11_ARATH Homeobox-leucine zipper protein
12	Solyc03g019820	Aquaporin	capana03g001527	CNG20_ARATH Probable cyclic nucleotide-gated ion channel 20
13	Solyc03g019810	Metallophosphoesterase	capana03g001528	SPTC2_HUMAN Serine palmitoyltransferase

14	Solyc03g019800	Myb-like protein B	capana03g001536	DPOE2_CHICK DNA polymerase epsilon subunit
15	Solyc03g019790	Alpha-galactosidase	capana03g001537	MYB44_ARATH Transcription factor MYB44
16	Solyc03g019780	40S ribosomal protein SA (eukaryotic/archaeal	capana03g001538	AGO1A_ORYSJ Protein argonaute 1A
17	Solyc03g019770	Uncharacterized PH domain-containing protein	capana03g001539	SUS2_PEA Sucrose synthase
18	Solyc03g019760	ABC transporter G family member 11	capana03g001540	DCOR_SOLLC Ornithine decarboxylase
19	Solyc03g019750	Alpha-1 4-glucan-protein synthase	capana03g001546	MYBG_DICDI Myb-like protein G
20	Solyc03g019740	Unknown Protein (AHRD V1)	capana03g001547	FBK6_ARATH Putative F-box/kelch-repeat protein
21	Solyc03g019730	Sumo activating enzyme 1b	capana03g001549	
22	Solyc03g019720	Carnitine operon protein caiE	capana03g001552	
23	Solyc03g019710	MADS-box transcription factor	capana03g001553	
24	Solyc03g019700	Os02g0508100 protein (Fragment) (AHRD V1 ***- Q0E0Z9_ORYSJ)		
25	Solyc03g019690	Kunitz-type protease inhibitor	capana03g001554	PP206_ARATH Putative pentatricopeptide repeat-containing protein
26	Solyc03g019680	CBS domain containing protein	capana06g000939	MORC3_HUMAN MORC family CW-type zinc finger protein
27	Solyc03g019670	Phosphoesterase family protein		
28	Solyc03g019660	Thylakoid lumenal 17.9 kDa protein%2C chloroplastic		
29	Solyc03g019650	Nodulin-like protein		
30	Solyc03g019640	Chaperone protein dnaJ 49		
31	Solyc03g019630	Chaperone protein dnaJ 3		
32	Solyc03g110840	Nuclear transcription factor Y subunit C-1	capana03g001462	UMP8_ARATH Uncharacterized protein
33	Solyc03g110850	Nuclear transcription factor Y subunit C-1		
34	Solyc03g110860	Nuclear transcription factor Y subunit C-1	capana03g001463	AGL5_ARATH Agamous-like MADS-box protein
35	Solyc03g110870	Unknown Protein (AHRD V1)		

36	Solyc03g110880	DNA-directed RNA polymerase	capana03g001461	SAE1B_ARATH SUMO-activating enzyme subunit 1B
37	Solyc03g110890	Expressed protein (Fragment)	capana03g001460	
38	Solyc03g110900	Katanin p60 ATPase-containing subunit A-like 1	capana03g001459	RGP3_ARATH UDP-arabinopyranose mutase 3
39	Solyc03g110910	Histone acetyltransferase ELP3 family protein	capana03g001458	AB11G_ARATH ABC transporter G family member
40	Solyc03g110920	Receptor expression-enhancing protein 5	capana03g001457	YBR6_SCHPO Uncharacterized protein
41	Solyc03g110930	30S ribosomal protein S21%2C chloroplastic (Fragment)	capana03g001456	RSSA_DAUCA 40S ribosomal protein SA
42	Solyc03g110940	Uncharacterized aarF domain-containing protein kinase 1	capana03g001451	AGAL_COFAR Alpha-galactosidase
43	Solyc03g110950	GRAS family transcription factor	capana03g001450	DMTF1_HUMAN Cyclin-D-binding Myb-like transcription factor 1
44	Solyc03g110960	Nucleoside diphosphate kinase	capana03g001449	
45	Solyc03g110970	GDP-mannose transporter	capana03g001448	TIP32_ARATH Probable aquaporin TIP3-2
46	Solyc03g110980	Unknown Protein (AHRD V1)	capana03g001447	Y1680_ARATH Probable leucine-rich repeat receptor-like protein kinase
47	Solyc03g110990	Unknown Protein (AHRD V1)		
48	Solyc03g111000	Glyceraldehyde 3-phosphate dehydrogenase	capana03g001445	
49	Solyc03g111010	Glyceraldehyde-3-phosphate dehydrogenase		
50	Solyc03g111020	Unknown Protein (AHRD V1)		
51	Solyc03g111030	Genomic DNA chromosome 5 P1 clone MXI22	capana03g001444	M2K6_ARATH Mitogen-activated protein kinase kinase 6
52	Solyc03g111040	Histidine amino acid transporter	capana03g001442	C72B1_PINTA Abietadienol/abietadienal oxidase
53	Solyc03g111050	Plastid RNA-binding protein	capana03g001397	RPD2A_ARATH DNA-directed RNA polymerase D subunit 2a
54	Solyc03g111060	3-oxoacyl-reductase	capana06g000949	BOR2_ARATH Probable boron transporter 2

55	Solyc03g111070	3-oxoacyl-reductase	capana03g001400	NFYC4_ARATH Nuclear transcription factor Y subunit C-4
56	Solyc03g111080	3-oxoacyl- reductase		
57	Solyc03g111090	Bromodomain factor	capana03g001404	
58	Solyc03g111100	AtIII18x5-like protein (Fragment)	capana03g001419	IP23_SOLLC Proteinase inhibitor type-2 CEVI57
59	Solyc03g111110	Unknown Protein (AHRD V1)		
60	Solyc03g111120	Malate synthase	capana03g001420	MIRA_RICDU Miraculin
61	Solyc03g111130	Malate synthase		
62	Solyc03g111140	Malate synthase		
63	Solyc03g111150	Extracellular ligand-gated ion channel	capana03g001422	
64	Solyc03g111160	Dual-specificity protein-like phosphatase 3		
65	Solyc03g111170	4-coumarate-coa ligase	capana03g001424	PP165_ARATH Pentatricopeptide repeat-containing protein

Table S10. Annotation of genes in *ty5* qtl region of Tomato and their conserved orthologs in pepper

S.No	Tomato gene	Annotation in Tomato	Pepper ortholog	Annotation in Pepper
1.	Solyc04g008510	UBX domain-containing protein		
2.	Solyc04g008520	Outer envelope protein (	capana05g000831	ARL8B_RAT ADP-ribosylation factor-like protein
3.	Solyc04g008530	Ubiquitin carboxyl-terminal hydrolase family protein	capana05g000818	HMGYA_SOYBN HMG-Y-related protein A
4.	Solyc04g008540	Tobamovirus multiplication protein (Fragment)	capana05g000819	XPC_MOUSE DNA repair protein complementing XP-C cells homolog
5.	Solyc04g008550	Zinc finger CCCH domain-containing protein 17	capana05g000820	RL23_METCA 50S ribosomal protein L23
6.	Solyc04g008560	Unknown Protein (AHRD V1)	capana05g000823	#N/A
7.	Solyc04g008570	Pentatricopeptide repeat-containing protein	capana03g001301	Pentatricopeptide repeat-containing protein
8.	Solyc04g008580	Ankyrin repeat domain protein (	capana05g000817	#N/A
9.	Solyc04g008590	Pyruvate dehydrogenase E1 component subunit beta	capana05g000815	Probably inactive leucine-rich repeat receptor-like protein kinase
10.	Solyc04g008600	Chromosome segregation in meiosis protein 3	capana05g000806	#N/A
11.	Solyc04g008610	Histone acetyltransferase	capana03g001296	BT1_ARATH BTB/POZ and TAZ domain-containing protein 1
12.	Solyc04g008620	Integral membrane single C2 domain protein	capana05g000800	GGPPS_HEVBR Geranylgeranyl pyrophosphate synthase, chloroplastic
13.	Solyc04g008630	Autophagy-related protein 9	capana05g000801	FDL1_ARATH F-box/FBD/LRR-repeat protein
14.	Solyc04g008640	Conserved oligomeric Golgi complex subunit 3		#N/A
15.	Solyc04g008650	Receptor like kinase%2C RLK		#N/A
16.	Solyc04g008660	Arf-GAP with GTPase%2C ANK repeat and PH domain-containing protein 1	capana05g000802	#N/A
17.	Solyc04g008670	Gibberellin 2-beta-dioxygenase 7	capana03g001342	KING1_ARATH SNF1-related protein kinase regulatory subunit gamma-1

18.	Solyc04g008680	Cell division cycle and apoptosis regulator protein 1 (	capana05g000805	GLYT4_ARATH Probable glycosyltransferase
19.	Solyc04g008690	cDNA clone J013057D02 full insert sequence	capana05g000798	GAI_SOLLC DELLA protein GAI
20.	Solyc04g008700	Pre-mRNA-processing factor 40 homolog A	capana05g000794	RHIE_DICD3 Rhamnogalacturonate lyase
21.	Solyc04g008710	Unknown Protein (AHRD V1)	capana05g000793	BPC1_ARATH Protein BASIC PENTACYSTEINE1
22.	Solyc04g008720	WD-repeat protein	capana03g001292	AGO4B_ORYSJ Protein argonaute 4B
23.	Solyc04g008730	Alpha-galactosidase 1	capana05g000781	IAR1_ARATH IAA-alanine resistance protein 1
24.	Solyc04g008740	Pyruvate kinase	capana05g000778	CAP13_ARATH Putative clathrin assembly protein
25.	Solyc04g008750	Transcription elongation factor SPT4	capana05g000768	Y1133_ARATH G-type lectin S-receptor-like serine/threonine-protein kinase
26.	Solyc04g008760	Homoserine kinase	capana05g000767	PPR6_ARATH Pentatricopeptide repeat-containing protein
27.	Solyc04g008770	Mitochondrial intermembrane space import and assembly protein	capana05g000766	MS1_ARATH PHD finger protein MALE STERILITY 1
28.	Solyc04g008780	Dihydroflavonol 4-reductase family	capana05g000764	BRL2_ARATH Serine/threonine-protein kinase BRI1-like 2
29.	Solyc04g008790	LOC563247 protein (Fragment)	capana05g000760	HAK17_ORYSJ Probable potassium transporter 17
30.	Solyc04g008800	Unknown Protein (AHRD V1)	capana05g000757	MYBF_ARATH Putative Myb family transcription factor
31.	Solyc04g008810	40S ribosomal protein S26	capana05g000755	OEP37_PEA Outer envelope pore protein 37, chloroplastic
32.	Solyc04g008820	HMG transcription factor (Fragment)	capana05g000754	UBXN7_PONAB UBX domain-containing protein 7
33.	Solyc04g008830	LRR receptor-like serine/threonine-protein kinase%2C RLP	capana05g000753	MGP_ARATH Zinc finger protein MAGPIE OS=Arabidopsis thaliana GN=MGP PE=1 SV=1
34.	Solyc04g008840	ABI3-interacting protein 2	capana05g000752	NDUB7_ARATH NADH dehydrogenase [ubiquinone] 1 beta subcomplex subunit 7

35.	Solyc04g008850	Aspartic proteinase nepenthesin-1	capana05g000748	PP379_ARATH Pentatricopeptide repeat-containing protein
36.	Solyc04g008860	Arginine N-methyltransferase family protein	capana05g000747	Y2215_ARATH Uncharacterized protein
37.	Solyc04g008870	MYB transcription factor	capana05g000746	Zinc finger CCCH domain-containing protein 17
38.	Solyc04g008880	UDP-N-acetylmuramoyl-tripeptide--D-alanyl-D-alanine ligase		#N/A
39.	Solyc04g008890	Splicing factor 3B subunit 5		#N/A
40.	Solyc04g008900	Fgenesh protein 60		#N/A
41.	Solyc04g008910	Fgenesh protein 60		#N/A
42.	Solyc04g008920	Fgenesh protein 60		#N/A
43.	Solyc04g008930	FIP1		#N/A
44.	Solyc04g008940	FIP1		#N/A
45.	Solyc04g008950	FIP1		#N/A
46.	Solyc04g008960	FIP1 (		#N/A
47.	Solyc04g008970	FIP1		#N/A
48.	Solyc04g008980	F-box/LRR-repeat protein		#N/A
49.	Solyc04g008990	Riboflavin biosynthesis protein RibF		#N/A
50.	Solyc04g009000	Pentatricopeptide repeat-containing protein		#N/A
51.	Solyc04g009010	Isoprenylcysteine carboxyl methyltransferase		#N/A
52.	Solyc04g009020	Pre-mRNA-splicing factor clf1		#N/A
53.	Solyc04g009030	Glyceraldehyde-3-phosphate dehydrogenase		#N/A
54.	Solyc04g009040	Receptor like kinase%2C RLK		#N/A
55.	Solyc04g009050	Os01g0498200 protein (Fragment)		#N/A
56.	Solyc04g009060	CD2 antigen cytoplasmic tail-binding protein 2		#N/A
57.	Solyc04g009070	Nbs%2C resistance protein fragment		#N/A
58.	Solyc04g009080	Nbs%2C resistance protein fragment		#N/A
59.	Solyc04g009090	Nbs-lrr%2C resistance protein		#N/A
60.	Solyc04g009100	Nbs%2C resistance protein fragment		#N/A
61.	Solyc04g009110	Cc-nbs-lrr%2C resistance protein		#N/A

62.	Solyc04g009120	Cc-nbs-lrr%2C resistance protein		#N/A
63.	Solyc04g009130	Cc-nbs-lrr%2C resistance protein		#N/A
64.	Solyc04g009140	Mitochondrial ribosomal protein L46		#N/A
65.	Solyc04g009150	Cc-nbs-lrr%2C resistance protein		#N/A
66.	Solyc04g009160	Unknown Protein (AHRD V1)		#N/A
67.	Solyc04g009170	RING finger protein 44	capana03g001962	WRK27_ARATH Probable WRKY transcription factor 27
68.	Solyc04g009180	TCP family transcription factor	capana03g001963	#N/A
69.	Solyc04g009190	WW domain-binding protein 2	capana03g001964	PP151_ARATH Pentatricopeptide repeat-containing protein
70.	Solyc04g009200	Glutamate-1-semialdehyde-2 1-aminomutase	capana03g001966	DOF46_ARATH Dof zinc finger protein
71.	Solyc04g009210	Pentatricopeptide repeat-containing protein	capana03g001967	MUT7_AEDAE Probable exonuclease mut-7 homolog
72.	Solyc04g009220	Pentatricopeptide repeat-containing protein		#N/A
73.	Solyc04g009230	Mitosis protein dim1	capana03g001971	ATXR6_ARATH Histone-lysine N-methyltransferase
74.	Solyc04g009240	Cc-nbs-lrr%2C resistance protein		#N/A
75.	Solyc04g009250	Cc-nbs-lrr%2C resistance protein		#N/A
76.	Solyc04g009260	Cc-nbs-lrr%2C resistance protein		#N/A
77.	Solyc04g009270	Cc-nbs%2C resistance protein fragment		#N/A
78.	Solyc04g009280	IMP dehydrogenase/GMP reductase (AHRD V1 **-- B6SV42_MAIZE)		#N/A
79.	Solyc04g009290	Cc-nbs-lrr%2C resistance protein		#N/A
80.	Solyc04g009300	GTP-binding protein YqeH		#N/A
81.	Solyc04g009310	ATP-dependent Clp protease proteolytic subunit		#N/A
82.	Solyc04g009320	Hsp70 nucleotide exchange factor fes1		#N/A
83.	Solyc04g009330	Serine/threonine-protein kinase 12		#N/A
84.	Solyc04g009340	U-box domain-containing protein 62		#N/A
85.	Solyc04g009350	Histidinol-phosphate aminotransferase		#N/A
86.	Solyc04g009360	WD-40 repeat family protein		#N/A
87.	Solyc04g009370	Homology to unknown gene		#N/A

88.	Solyc04g009380	Palmitoyltransferase PFA4 Zinc finger%2C DHHC-type		#N/A
89.	Solyc04g009390	PHD-finger family protein expressed		#N/A
90.	Solyc04g009400	PHD-finger family protein expressed		#N/A
91.	Solyc04g009410	Proteasome subunit beta type		#N/A
92.	Solyc04g009420	Photosystem II oxygen evolving complex protein PsbP		#N/A
93.	Solyc04g009430	NAD-dependent		#N/A
94.	Solyc04g009440	NAC domain protein		#N/A
95.	Solyc04g009450	Ethylene-responsive transcription factor 4		#N/A
96.	Solyc04g009460	Unknown Protein		#N/A
97.	Solyc04g009470	CTV.22	capana05g000745	#N/A
98.	Solyc04g009480	Unknown Protein		#N/A
99.	Solyc04g009490	CTV.22	capana05g000743	UBP12_ARATH Ubiquitin carboxyl-terminal hydrolase 12
100.	Solyc04g009500	CTV.22		#N/A
101.	Solyc04g009510	CCR4-NOT transcription complex subunit 4	capana05g000741	GABP2_BOVIN GA-binding protein subunit beta-2
102.	Solyc04g009520	Nuclear transcription factor Y subunit B-3	capana05g000740	ODPB_ZYGCR Pyruvate dehydrogenase E1 component subunit beta
103.	Solyc04g009530	Glutathione S-transferase	capana05g000733	TIPIN_BOVIN TIMELESS-interacting protein
104.	Solyc04g009540	50S ribosomal protein L10	capana05g000711	CCAR1_HUMAN Cell division cycle and apoptosis regulator protein 1
105.	Solyc04g009550	FGFR1 oncogene partner	capana05g000709	G2OX8_ARATH Gibberellin 2-beta-dioxygenase 8
106.	Solyc04g009560	TBC1 domain family member 8B	capana05g000708	AGD4_ARATH ADP-ribosylation factor GTPase-activating protein AGD4
107.	Solyc04g009570	Chromosome 15 contig 1 DNA sequence		#N/A
108.	Solyc04g009580	CDP-diacylglycerol--glycerol-3-phosphate 3-phosphatidyltransferase	capana05g000706	ATG9_DICDI Autophagy-related protein 9
109.	Solyc04g009590	Defensin protein		#N/A

110.	Solyc04g009600	TRAF-type zinc finger family protein	capana05g000705	ESYT2_MOUSE Extended synaptotagmin-2
111.	Solyc04g009610	Fasciclin-like domain-containing protein	capana05g000696	Histone acetyltransferase
112.	Solyc04g009620	Chorismate synthase	capana05g000685	#N/A
113.	Solyc04g009630	Alpha-glucosidase 1	capana05g000681	TCRG1_MOUSE Transcription elongation regulator 1
114.	Solyc04g009640	Receptor like kinase%2C RLK	capana05g000680	#N/A
115.	Solyc04g009650	Diphosphomevalonate decarboxylase-like protein		#N/A
116.	Solyc04g009660	Cc-nbs-lrr%2C resistance protein	capana05g000653	NEP2_NEPGR Aspartic proteinase nepenthesin-2
117.	Solyc04g009670	Acyltransferase (Fragment)		#N/A
118.	Solyc04g009680	Acyltransferase (Fragment)		#N/A
119.	Solyc04g009690	Cc-nbs-lrr%2C resistance protein		#N/A
120.	Solyc04g009700	Dual specificity phosphatase catalytic domain containing protein expressed	capana05g000678	KTNB1_ARATH Katanin p80 WD40 repeat-containing subunit B1 homolog 1
121.	Solyc04g009710	Ferredoxin	capana05g000672	AGAL_COFAR Alpha-galactosidase
122.	Solyc04g009720	Pentatricopeptide repeat-containing protein	capana05g000670	KPYC_SOLTU Pyruvate kinase, cytosolic isozyme
123.	Solyc04g009730	Pentatricopeptide repeat-containing protein	capana12g002230	MVD1_DICDI Diphosphomevalonate decarboxylase
124.	Solyc04g009740	Exocyst complex protein EXO70	capana05g000669	SPT42_ARATH Transcription elongation factor SPT4 homolog
125.	Solyc04g009750	Unknown Protein (AHRD V1)		#N/A
126.	Solyc04g009760	AT5G22070 protein (Fragment)	capana05g000668	KHSE_PROVI Homoserine kinase
127.	Solyc04g009770	DNAJ chaperone	capana05g000666	MIA40_USTMA Mitochondrial intermembrane space import and assembly protein 40
128.	Solyc04g009780	RING finger protein 13	capana05g000665	DFRA_MALDO Bifunctional dihydroflavonol 4-reductase/flavanone 4-reductase
129.	Solyc04g009790	Multidrug resistance protein mdtK		#N/A
130.	Solyc04g009800	Calcium-dependent protein kinase 2	capana05g000652	ANM6_ARATH Probable protein arginine

				N-methyltransferase 6
131.	Solyc04g009810	Pelota homolog	capana05g000663	#N/A
132.	Solyc04g009820	Calcium-responsive transactivator	capana05g000659	RS261_ARATH 40S ribosomal protein S26-1
133.	Solyc04g009830	Stress responsive gene 6 protein Srg6	capana05g000658	HMGB7_ARATH High mobility group B protein 7
134.	Solyc04g009840	C-Myc-binding protein	capana05g000654	FRIGI_ARATH Protein FRIGIDA
135.	Solyc04g009850	1-aminocyclopropane-1-carboxylate oxidase-like protein		#N/A
136.	Solyc04g009860	1-aminocyclopropane-1-carboxylate oxidase-like protein		#N/A
137.	Solyc04g009870	Unknown Protein		#N/A
138.	Solyc04g009880	Unknown Protein		#N/A
139.	Solyc04g009890	Unknown Protein		#N/A
140.	Solyc04g009900	Calcium-dependent protein kinase 2		#N/A
141.	Solyc04g009910	Calcium-dependent protein kinase 2		#N/A
142.	Solyc04g009920	Glycogenin-like protein	capana05g000644	MY1R1_SOLTU Transcription factor MYB1R1
143.	Solyc04g009930	Cytochrome P450	capana05g000548	#N/A
144.	Solyc04g009940	Heat shock protein binding protein		#N/A
145.	Solyc04g009950	Pre-mRNA splicing factor	capana05g000547	#N/A
146.	Solyc04g009960	L-allo-threonine aldolase	capana05g000546	NOT4_YEAST General negative regulator of transcription subunit 4
147.	Solyc04g009970	Zinc ion binding protein	capana05g000545	NFYB8_ARATH Nuclear transcription factor Y subunit B-8
148.	Solyc04g009980	Light-dependent short hypocotyls 1	capana05g000544	IN21B_ORYSJ Protein IN2-1 homolog B
149.	Solyc04g009990	Lysine-specific demethylase 5A	capana05g000543	RK10_TOBAC 50S ribosomal protein L10, chloroplastic
150.	Solyc04g010000	RNA-binding protein	capana05g000542	FR1OP_HUMAN FGFR1 oncogene partner
151.	Solyc04g010010	Thioredoxin-like protein 1		#N/A
152.	Solyc04g010020	RNA-binding protein		#N/A

153.	Solyc04g010030	Thioredoxin-like protein 1		#N/A
154.	Solyc04g010040	RNA-binding protein		#N/A
155.	Solyc04g010050	Pentatricopeptide repeat-containing protein	capana05g000541	GYP2_EMENI Putative GTPase-activating protein AN11010
156.	Solyc04g010060	Pentatricopeptide repeat-containing protein		#N/A
157.	Solyc04g010070	Protein phosphatase 2A regulatory subunit B%26apos-like protein	capana05g000539	PGSA_SODGM CDP-diacylglycerol--glycerol-3-phosphate 3-phosphatidyltransferase
158.	Solyc04g010080	ATP-binding domain-containing protein 4	capana05g000538	#N/A
159.	Solyc04g010090	Transcription initiation factor TFIID subunit 9B	capana05g000533	#N/A
160.	Solyc04g010100	50S ribosomal protein L33		#N/A
161.	Solyc04g010110	Anthocyanidin 3-O-glucosyltransferase	capana05g000531	AROC2_SOLLC Chorismate synthase 2, chloroplastic
162.	Solyc04g010120	Macrophage erythroblast attacher	capana05g000518	AGLU_SPIOL Alpha-glucosidase
163.	Solyc04g010130	Unknown Protein (AHRD V1)	capana05g000517	Y3475_ARATH Probable LRR receptor-like serine/threonine-protein kinase
164.	Solyc04g010140	Unknown Protein (AHRD V1)		#N/A
165.	Solyc04g010150	Unknown Protein (AHRD V1)		#N/A
166.	Solyc04g010160	Unknown Protein (AHRD V1)		#N/A
167.	Solyc04g010180	Unknown Protein (AHRD V1)	capana05g000516	Uncharacterized protein ynbD
168.	Solyc04g010190	Immunophilin-like	capana05g000515	FER6_MAIZE Ferredoxin-6, chloroplastic
169.	Solyc04g010200	ABC transporter G family member 6	capana05g000514	PP365_ARATH Pentatricopeptide repeat-containing protein
170.	Solyc04g010210	ABC transporter G family member 1		#N/A
171.	Solyc04g010220	Unknown Protein (AHRD V1)	capana05g000513	EXOC7_RAT Exocyst complex component 7
172.	Solyc04g010230	Pectate lyase family protein	capana05g000508	#N/A
173.	Solyc04g010240	60S ribosomal protein L35	capana05g000506	DNJH2_ALLPO DnaJ protein homolog 2
174.	Solyc04g010250	Lipase-like protein	capana05g000503	ATL71_ARATH Putative RING-H2 finger protein
175.	Solyc04g010260	Pre-mRNA-splicing factor RSE1	capana05g000502	CDPK1_ARATH Calcium-dependent

				protein kinase 1
176.	Solyc04g010270	Disease resistance response/ dirigent-like protein	capana05g000501	PELO_DROME Protein pelota
177.	Solyc04g010280	Unknown Protein (AHRD V1)		#N/A
178.	Solyc04g010290	Beta-1-3-galactosyl-o-glycosyl-glycoprotein	capana05g000500	CREST_XENLA Calcium-responsive transactivator 1
179.	Solyc04g010300	Unknown Protein (AHRD V1)	capana05g000499	VPS72_BOVIN Vacuolar protein sorting-associated protein 72 homolog
180.	Solyc04g010310	Uncharacterized ABC transporter ATP-binding protein/permease C9B6.09c		#N/A
181.	Solyc04g010320	Cytochrome P450	capana05g000498	ZOG_PHALU Zeatin O-glucosyltransferase
182.	Solyc04g010330	Auxin-regulated protein		#N/A
183.	Solyc04g011330	Unknown Protein		#N/A
184.	Solyc04g011340	Nodulin-like protein	capana05g000461	R1B16_SOLDE Putative late blight resistance protein homolog
185.	Solyc04g011350	2-oxoglutarate dehydrogenase E1 component	capana05g000457	PPCK1_ARATH Phosphoenolpyruvate carboxylase kinase 1
186.	Solyc04g011360	Ras-related protein Rab-8A	capana05g000409	XYLT_CIOSA Xylosyltransferase
187.	Solyc04g011370	Actin-depolymerizing factor 2	capana05g000408	AB20B_ARATH ABC transporter B family member 20
188.	Solyc04g011380	Binding protein	capana05g000407	C89A9_ARATH Cytochrome P450 89A9
189.	Solyc04g011390	Histone H4		#N/A
190.	Solyc04g011400	UDP-glucose 4-epimerase	capana05g000404	FBK50_ARATH F-box/kelch-repeat protein
191.	Solyc04g011420	UPF0497 membrane protein 2	capana05g000401	5NG4_PINTA Auxin-induced protein 5NG4
192.	Solyc04g011430	Ubiquitin-conjugating enzyme 13 E2	capana05g000399	ODO1_DICDI 2-oxoglutarate dehydrogenase, mitochondrial
193.	Solyc04g011440	heat shock protein		#N/A
194.	Solyc04g011450	Heat shock cognate protein 2		#N/A
195.	Solyc04g011460	SLT1 protein	capana05g000398	ELP1_RAT Elongator complex protein 1
196.	Solyc04g011470	Unknown Protein (AHRD V1)	capana05g000396	SYM_SYNY3 Methionine--tRNA ligase
197.	Solyc04g011480	UPF0497 membrane protein 17	capana05g000397	#N/A

198.	Solyc04g011490	UPF0497 membrane protein 17		#N/A
199.	Solyc04g011500	Actin 4	capana05g000395	UXS1_HUMAN UDP-glucuronic acid decarboxylase 1
200.	Solyc04g011510	Triosephosphate isomerase	capana05g000394	YN02_CAEEL CRAL-TRIO domain-containing protein T23G5.2
201.	Solyc04g011520	Serine/threonine kinase-like protein ABC1063	capana05g000393	CSPLI_POPTR CASP-like protein POPTRDRAFT_822486
202.	Solyc04g011530	Os04g0129600 protein (Fragment)	capana05g000392	UBC7_ARATH Ubiquitin-conjugating enzyme E2 7
203.	Solyc04g011540	Elicitor-responsive protein 1		#N/A
204.	Solyc04g011550	Unknown Protein	capana05g000391	PGTB1_RAT Geranylgeranyl transferase type-1 subunit beta
205.	Solyc04g011560	Heterogeneous nuclear ribonucleoprotein A3-like protein 2	capana05g000390	DOF36_ARATH Dof zinc finger protein DOF3.6
206.	Solyc04g011570	Zinc finger C3HC4 type family protein	capana05g000388	KIN17_MOUSE DNA/RNA-binding protein KIN17
207.	Solyc04g011580	mRNA binding protein Pumilio 2	capana05g000387	TECR_DICDI Trans-2,3-enoyl-CoA reductase
208.	Solyc04g011590	Histidine amino acid transporter	capana05g000386	ACT3_SOLTU Actin-58
209.	Solyc04g011600	ER glycerol-phosphate acyltransferase	capana05g000385	ALG5_HUMAN Dolichyl-phosphate beta-glucosyltransferase
210.	Solyc04g011610	RING finger protein 13	capana05g000384	#N/A
211.	Solyc04g011620	FK506-binding protein 5(PEPTIDYL-PROLYL CIS-TRANS ISOMERASE)		#N/A
212.	Solyc04g011630	GRAS family transcription factor	capana05g000382	PSF2_ARATH DNA replication complex GINS protein PSF2
213.	Solyc04g011640	Unknown Protein (AHRD V1)		#N/A
214.	Solyc04g011650	Telomere repeat-binding protein 4	capana05g000383	E13B_WHEAT Glucan endo-1,3-beta-glucosidase
215.	Solyc04g011660	Ulp1 protease family C-terminal catalytic domain containing protein		#N/A
216.	Solyc04g011670	BZIP transcription factor	capana05g000379	#N/A

217.	Solyc04g011680	Cytochrome P450		#N/A
218.	Solyc04g011690	Cytochrome P450		#N/A
219.	Solyc04g011700	Mitochondrial transcription termination factor family protein	capana05g000378	TRP4_ARATH Telomere repeat-binding protein 4
220.	Solyc04g011710	Unknown Protein (AHRD V1)		#N/A
221.	Solyc04g011720	Glucan endo-1 3-beta-glucosidase 5	capana05g000374	GAT15_ARATH GATA transcription factor 15
222.	Solyc04g011730	Glucan endo-1 3-beta-glucosidase 5		#N/A
223.	Solyc04g011740	Unknown Protein (AHRD V1)	capana05g000373	ARR2_ARATH Two-component response regulator ARR2
224.	Solyc04g011750	Unknown Protein (AHRD V1)		#N/A
225.	Solyc04g011760	Unknown Protein (AHRD V1)		#N/A
226.	Solyc04g011770	Unknown Protein (AHRD V1)		#N/A
227.	Solyc04g011780	Glutaredoxin	capana05g000372	PRS2_SOLTU Pathogenesis-related protein STH-2
228.	Solyc04g011790	Glutaredoxin		#N/A
229.	Solyc04g011800	Glutaredoxin		#N/A
230.	Solyc04g011810	Glutaredoxin		#N/A
231.	Solyc04g011820	Glutaredoxin		#N/A
232.	Solyc04g011830	Glutaredoxin		#N/A
233.	Solyc04g011840	Glutaredoxin		#N/A
234.	Solyc04g011850	Glutaredoxin		#N/A
235.	Solyc04g011860	Glutaredoxin		#N/A
236.	Solyc04g011870	Glutaredoxin		#N/A
237.	Solyc04g011880	Glutaredoxin		#N/A
238.	Solyc04g011890	Nbs%2C resistance protein fragment		#N/A
239.	Solyc04g011900	Os04g0639300 protein		#N/A
240.	Solyc04g011910	Os03g0169900 protein		#N/A
241.	Solyc04g011920	Cytochrome P450		#N/A
242.	Solyc04g011940	Cytochrome P450		#N/A

243.	Solyc04g011950	Unknown Protein (AHRD V1)		#N/A
244.	Solyc04g011960	Cc-nbs-lrr%2C resistance protein		#N/A
245.	Solyc04g011970	Gag-Pol polyprotein		#N/A
246.	Solyc04g011980	Cc-nbs-lrr%2C resistance protein		#N/A
247.	Solyc04g011990	Cc-nbs-lrr%2C resistance protein		#N/A
248.	Solyc04g012000	Nbs%2C resistance protein fragment		#N/A
249.	Solyc04g012010	Cc-nbs-lrr%2C resistance protein		#N/A
250.	Solyc04g012020	Hydroxycinnamoyl CoA quinate transferase		#N/A
251.	Solyc04g012030	Palmitoyltransferase PFA4		#N/A
252.	Solyc04g012040	26S proteasome non-ATPase regulatory subunit 12		#N/A
253.	Solyc04g012050	Ethylene responsive transcription factor 2a		#N/A
254.	Solyc04g012060	Ribonucleoside-diphosphate		#N/A
255.	Solyc04g012070	Nodulin family protein		#N/A
256.	Solyc04g012080	Pentatricopeptide repeat-containing protein		#N/A
257.	Solyc04g012090	Receptor like kinase%2C RLK		#N/A
258.	Solyc04g012100	Receptor like kinase%2C RLK		#N/A
259.	Solyc04g012110	Receptor like kinase%2C RLK		#N/A
260.	Solyc04g012120	14-3-3 protein beta/alpha-1		#N/A
261.	Solyc04g012130	Gag-pol polyprotein		#N/A
262.	Solyc04g012140	Unknown Protein (AHRD V1)		#N/A
263.	Solyc04g012150	Unknown Protein (AHRD V1)		#N/A
264.	Solyc04g012160	Serine-threonine protein kinase		#N/A
265.	Solyc04g012170	Kinase family protein		#N/A
266.	Solyc04g012180	Ras-related protein Rab-25		#N/A
267.	Solyc04g012190	Receptor like kinase%2C RLK		#N/A
268.	Solyc04g012200	Unknown Protein (AHRD V1)		#N/A
269.	Solyc04g013200	Unknown Protein		#N/A
270.	Solyc04g014200	Receptor kinase-like protein		#N/A
271.	Solyc04g014210	ATP dependent RNA helicase		#N/A

272.	Solyc04g014220	RING finger protein		#N/A
273.	Solyc04g014230	Plant-specific domain TIGR01570 family protein		#N/A
274.	Solyc04g014240	Unknown Protein (AHRD V1)		#N/A
275.	Solyc04g014250	FIP1 (AHRD V1 **-- B6SNS7_MAIZE)%3B contains Interpro domain(s) IPR004182 GRAM		#N/A
276.	Solyc04g014260	Zinc finger-homeodomain protein 1		#N/A
277.	Solyc04g014270	Phosphofructokinase family protein		#N/A
278.	Solyc04g014280	Unknown Protein	capana05g000367	ACD10_MOUSE Acyl-CoA dehydrogenase family member 10 SV=1
279.	Solyc04g014290	Unknown Protein		#N/A
280.	Solyc04g014300	Unknown Protein		#N/A
281.	Solyc04g014310	Unknown Protein		#N/A
282.	Solyc04g014320	Unknown Protein		#N/A
283.	Solyc04g014330	Unknown Protein		#N/A
284.	Solyc04g014340	Unknown Protein		#N/A
285.	Solyc04g014350	Unknown Protein		#N/A
286.	Solyc04g014360	Transcription factor SPATULA	capana05g000366	PME28_ARATH Putative pectinesterase/pectinesterase inhibitor 28
287.	Solyc04g014370	Tyrosine phosphatase-like	capana05g000365	HYES_PIG Epoxide hydrolase 2
288.	Solyc04g014380	Kinase interacting protein 1	capana05g000364	Ras-related protein RABE1c
289.	Solyc04g014390	Mitochondrial ribosomal protein L43		#N/A
290.	Solyc04g014400	LRR receptor-like serine/threonine-protein kinase%2C RLP		#N/A
291.	Solyc04g014410	Serine/threonine protein kinase-like		#N/A
292.	Solyc04g014420	Receptor expression-enhancing protein 5		#N/A
293.	Solyc04g014430	Rhamnogalacturonate lyase		#N/A
294.	Solyc04g014440	LG127/30 like gene		#N/A
295.	Solyc04g014450	Rhamnogalacturonate lyase		#N/A
296.	Solyc04g014460	RNA binding protein	capana05g000363	ADF2_PETHY Actin-depolymerizing factor

				2
297.	Solyc04g014470	MYB transcription factor	capana05g000361	#N/A
298.	Solyc04g014480	class I heat shock protein 3	capana05g000358	UXS1_HUMAN UDP-glucuronic acid decarboxylase 1
299.	Solyc04g014490	Erwinia induced protein 2	capana05g000356	CSPLI_POPTR CASP-like protein POPTRDRAFT_822486
300.	Solyc04g014500	Diphosphoinositol polyphosphate phosphohydrolase	capana05g000355	UBC7_ARATH Ubiquitin-conjugating enzyme E2 7
301.	Solyc04g014510	Glutamine synthetase	capana05g000354	#N/A
302.	Solyc04g014520	Plant viral-response family protein		#N/A
303.	Solyc04g014530	Ethylene responsive transcription factor 1a	capana05g000351	PIMP1_CAPAN CASP-like protein PIMP1
304.	Solyc04g014540	Zinc finger protein 7	capana05g000349	PIMP1_CAPAN CASP-like protein PIMP1
305.	Solyc04g014550	Targeting protein for Xklp2 containing protein expressed		#N/A
306.	Solyc04g014560	Pre-mRNA-splicing factor 38B	capana05g000347	ACT11_SOLTU Actin-97
