# Supplementary figures and images for: Conservation and Dispersion of Genes Conferring Resistance to Tomato Begomoviruses between Tomato and Pepper Genomes
Source: Front Plant Sci. 2017 Nov 7;8:1803. doi: 10.3389/fpls.2017.01803 (PMC5681951; doi:10.3389/fpls.2017.01803)

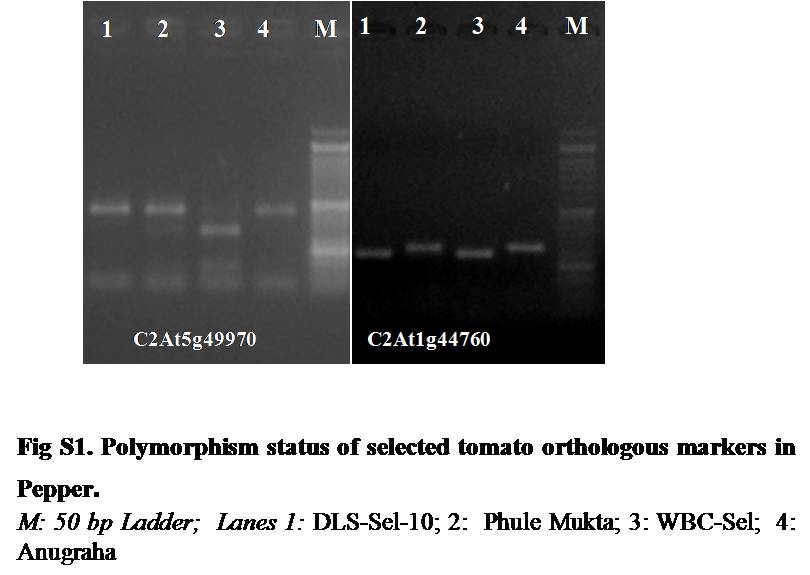

Supplement: Supplementary file 3 [file Image1.JPEG]
